# Dual roles of the conditional extracellular vesicles derived from *Pseudomonas aeruginosa* biofilms: Promoting and inhibiting bacterial biofilm growth

**DOI:** 10.1016/j.bioflm.2024.100183

**Published:** 2024-02-06

**Authors:** Marwa Gamal Saad, Haluk Beyenal, Wen-Ji Dong

**Affiliations:** The Gene and Linda Voiland School of Chemical Engineering and Bioengineering, Washington State University, Pullman, WA, 99164, USA

**Keywords:** Bacterial extracellular vesicles, Biofilm, *Pseudomonas aeruginosa,* biofilm control

## Abstract

Antibiotic-resistant biofilm infections have emerged as public health concerns because of their enhanced tolerance to high-dose antibiotic treatments. The biofilm life cycle involves multiple developmental stages, which are tightly regulated by active cell-cell communication via specific extracellular signal messengers such as extracellular vesicles. This study was aimed at exploring the roles of extracellular vesicles secreted by *Pseudomonas aeruginosa* at different developmental stages in controlling biofilm growth. Our results show that extracellular vesicles secreted by *P. aeruginosa* biofilms during their exponential growth phase (G-EVs) enhance biofilm growth. In contrast, extracellular vesicles secreted by *P. aeruginosa* biofilms during their death/survival phase (D-EVs) can effectively inhibit/eliminate *P. aeruginosa* PAO1 biofilms up to 4.8-log_10_ CFU/cm^2^. The inhibition effectiveness of D-EVs against *P. aeruginosa* biofilms grown for 96 h improved further in the presence of 10–50 μM Fe^3+^ ions. Proteomic analysis suggests the inhibition involves an iron-dependent ferroptosis mechanism. This study is the first to report the functional role of bacterial extracellular vesicles in bacterial growth, which depends on the developmental stage of the parent bacteria. The finding of D-EV-activated ferroptosis-based bacterial death may have significant implications for preventing antibiotic resistance in biofilms.

## Highlights

• Regulatory roles of bacterial extracellular vesicles in biofilm growth are culture condition dependent.
• D-EVs can prevent biofilm formation and treat pre-existing biofilms.
• Ferric ions enhance the power of the D-EVs in treating resilient aged biofilms.
• Ferroptosis may be involved in the action of the D-EVs against biofilms.

## Abbreviations

**PAO1**: *P. aeruginosa* PAO1
**G-EVs**: extracellular vesicles secreted by *P. aeruginosa* biofilms during their exponential growth phase
**D-EVs**: extracellular vesicles secreted by *P. aeruginosa* biofilms during their death/survival phase
**CLSM**: confocal laser scanning microscopy
**TEM**: transmission electron microscopy

## 1 Introduction

Drug-resistant bacterial infections are a significant and growing challenge to global health. Several gram-positive and gram-negative bacterial strains, including *Pseudomonas aeruginosa* and methicillin-resistant *Staphylococcus aureus* (MRSA), have been identified as drug-resistant pathogens, or "superbugs." In 2019, drug-resistant bacterial infections caused 1.27 million deaths [2], and this number is projected to increase to 10 million annual deaths worldwide by 2050 [3]. The existing antibiotics are often not effective enough at eradicating pathogenic bacteria. Antibiotic resistance becomes especially problematic when the pathogenic cells grow as biofilms [4]. Approximately 80% of drug-resistant bacterial infections in the human body are due to biofilms, and bacteria within these biofilms can increase their antibiotic resistance up to 1000-fold [5].

Biofilm development involves multiple stages, including attachment, adaptation followed by exponential growth, maturation, stationary growth, and dispersion leading to new biofilm formation. These stages can be influenced by the culture environment, with some stress or hostile culture conditions forcing the bacterial biofilm into a cell death/survival phase, in which bacterial populations can constantly switch between growth, survival, and death [6]. Each of these developmental stages is tightly regulated by active cell-cell communication via various signaling mediators, including extracellular vesicles (EVs) to coordinate cellular processes [[7], [8], [9]].

Extracellular vesicles are membrane-bound nanovesicles with diameters of 30–400 nm that can be secreted by all types of cells. They transfer lipids, proteins, mRNAs, and microRNAs from parental cells to other cells, thus altering the target cells' behavior, and making extracellular vesicles important mediators of intercellular communication [[10], [11], [12]]. Extracellular vesicle-based intercellular communication has been extensively studied in many biological and pathological processes, particularly in the context of cancer [[13], [14], [15]], but it is now recognized as a primordial feature of all living cells [16,17]. In recent years, it has been recognized that the secretion of extracellular vesicles appears to be a conserved process in both gram-negative and gram-positive bacteria. For example, the human pathogen *Pseudomonas aeruginosa*, an important Gram-negative bacterium and a major cause of infectious keratitis, uses EVs as a part of its signal trafficking system to mediate cell-cell communications within a bacterial community and coordinate group behaviors of the bacterial population [18]. Other studies suggest that bacterial EVs are also involved in regulating essential cellular processes of bacterial life cycles [7,8], including cellular division, the formation and maintenance of biofilms [9], and the transferring of DNA to other bacteria sharing genes involved in antibiotic resistance [19,20]. We hypothesized that the role of bacterial EVs in biofilm development is culture condition dependent, i.e., that the EVs secreted by biofilms in their exponential growth phase and their survival/death phase would promote biofilm growth and inhibit biofilm growth/formation, respectively.

To test our hypothesis, we investigated whether EVs derived from Gram-negative *P. aeruginosa* (PAO1) biofilms at different developmental stages can be utilized to control biofilm formation and development. These stage-dependent EVs are referred to as conditional bacterial extracellular vesicles. Briefly, we extracted EVs released by PAO1 biofilms at their exponential growth stage (G-EVs) and their survival/death phase (D-EVs), respectively. We then examined how these conditional EVs control PAO1 biofilm growth under different conditions. The results of our study demonstrate the potential of conditional bacterial EVs as alternative antibiotic agents for addressing the issue of antibiotic resistance currently facing society.

## 2 Results

### 2.1 Isolation and characterization of conditional extracellular vesicles secreted by *P. aeruginosa* PAO1 biofilms

**Fig. 1:**
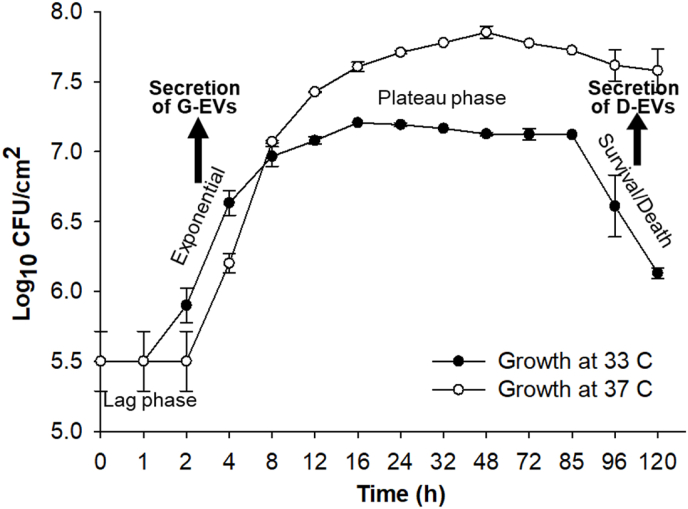
Growth curves of *P. aeruginosa* PAO1 biofilms inoculated and incubated at 33 °C and 37 °C, respectively, on TSA plates. Four stages of biofilm growth are clearly presented for the growth curve of 33 °C. Arrows show the stages where the biofilms were collected to extract G-EVs and D-EVs, respectively. Note: the scale of the x-axis is not linear. Data points indicate mean logCFU/cm^2^, and error bars indicate standard deviations (n = 4).

Fig. 1 shows *P. aeruginosa* PAO1 biofilm growth curves at 33 °C and 37 °C. The growth curve for 33 °C looks similar to a typical growth curve observed for shaken or well-mixed planktonic cells. When growing at 37 °C, *P. aeruginosa* PAO1 biofilm reached a steady cell number of ∼7.7 Log_10_ CFU/cm^2^ at the 16th hour and the plateau phase lasted until the 120th hour. However, the survival/death phase was not distinguishable before the 120th hour for biofilms grown at 37 °C. Under a 33 °C culture condition, PAO1 biofilm growth clearly showed four growth stages: lag, exponential growth, plateau, and survival/death, with a steady cell number of ∼6 Log_10_ CFU/cm^2^ in the plateau phase. Based on these observations, in this study, all conditional extracellular vesicles were extracted from *P. aeruginosa* PAO1 biofilms cultured under the 33 °C condition. However, all functional tests of the obtained extracellular vesicles on *P. aeruginosa* PAO1 biofilm growth were performed under the 37 °C culture condition. We should note that the colony biofilm model we used should have heterogeneity. For example, the cells in the center may be less metabolically active or dying, while at the edge, metabolic activity is expected to be higher. Since we quantified the average viable cell numbers, the growth curve given in Fig. 1 represents the physiological state of the majority of the cells.

**Fig. 2:**
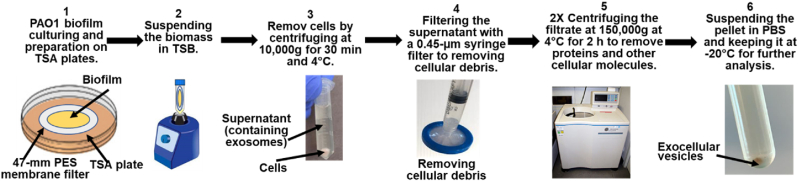
The extracellular vesicle extraction protocol consisted of 6 major steps. 1) Culturing PAO1 biofilm on filter membrane: *P. aeruginosa* PAO1 bacterial inoculum with 4.4 × 10^5^ ± 7.3 × 10^2^ CFU/cm^2^ was used to seed a 47-mm sterilized membrane on sterilized TSA plates and incubated in the dark at 33 °C until biofilms reached either exponential growth or survival/death phases. 2) Suspending and breaking up the biofilms to release extracellular vesicles. 3) Spinning down the bacterial cells. 4) Filtering the supernatant through a 0.45-μm filter to remove cellular debris. 5) Centrifuging at ultrahigh speed to isolate EVs from soluble proteins and other cellular molecules. After the EV pellet was resuspended in PBS buffer, the sample was filtered with a 0.22-μm syringe filter further to remove small cellular debris before the second ultrahigh-speed centrifugation to pellet down the target EVs. 6) Suspending the EV pellet in PBS buffer and keeping the extracted extracellular vesicles at −20 °C for further analysis.

G-EVs or D-EVs of *P. aeruginosa* PAO1 biofilms were extracted from biofilms grown at 33 °C. The biofilms were collected at specific developmental stages, as indicated in Fig. 1, using conventional differential centrifugation protocols [[21], [22], [23], [24]], which are given in the Materials and Methods section and summarized in Fig. 2. Using this protocol, a total of 874.1 ± 20 μg extracellular vesicle proteins could be extracted from membrane biofilms with an initial cell concentration of 4.4 × 10^5^ ± 7.3 × 10^2^ CFU/cm^2^.

The protein concentration of the D-EVs and the number of D-EV particles in a solution sample were correlated using stained extracellular vesicle samples and known protein concentrations (see Supplementary Information section S1). The results (shown in Fig. S1 and Fig. S2) established the correlation between the protein concentrations of the D-EVs and their average particle numbers in a solution, with every D-EV particle equivalent to an average extracellular vesicle protein content of 0.00188 ± 8 × 10^−4^ μg/ml.

The shapes and sizes of the purified G-EVs and D-EVs were characterized using transmission electron microscopy (TEM) analysis (see Supplementary Information section S2 and Fig. S3). The size of extracellular vesicles extracted during the exponential growth phase was larger (112.9 ± 3.7 nm) than that of extracellular vesicles extracted during the death/survival phase (33.2 ± 0.9 nm). These results demonstrate that *P. aeruginosa* PAO1 biofilms release different populations of extracellular vesicles at different stages, consistent with results observed for tumor cells and HeLa cells, which release different populations and subpopulations of EVs [25,26]. This suggests that these distinct populations of extracellular vesicles secreted by *P. aeruginosa* PAO1 biofilms at different stages have different functional roles in the biofilm life cycle.

### 2.2 The functional effects of G-EVs and D-EVs on the growth of *P. aeruginosa* PAO1 biofilm

To investigate our hypothesis that extracellular vesicles produced by a bacterial biofilm at a specific growth stage can influence the growth of recipient bacterial communities in the same developmental direction, we conducted a disc diffusion assay using both D-EVs and G-EVs to examine their functional effects on the growth behavior of *P. aeruginosa* PAO1 biofilm (see *Supplementary Information section S3* and Fig. S4). We used PBS buffer and tetracycline as negative and positive controls, respectively, to assess the efficacy of D-EVs/G-EVs in inhibiting/promoting the formation and growth of *P. aeruginosa* PAO1 biofilms in comparison to tetracycline. Given that *P. aeruginosa* PAO1 is a known antibiotic-resistant superbug, we used a sub-inhibitory concentration of tetracycline (1 μg/μL) in the test to ensure a clear zone on the dish (Fig. S4A). This concentration is much higher than the medically tolerated doses (0.033–0.22 μg/μL) used for adult admissions [1]. Although the qualitative nature of the diffusion test made it difficult to discern the role of G-EVs in biofilm formation and growth, our D-EVs exhibited an effective and dose-dependent inhibition of *P. aeruginosa* PAO1 biofilm growth across a range of extracellular vesicle protein concentrations (0.11, 0.22, and 0.33 μg/μL).

In order to confirm the importance of intact extracellular vesicle structure for the observed effects, we conducted an experiment in which we disrupted the membranes of D-EVs and tested whether this affected their ability to inhibit biofilm growth. We achieved this by subjecting the D-EV sample to water bath sonication, which lysed the extracellular vesicles (see Supplementary Information section S3). We then used the sonicated D-EVs in the diffusion susceptibility test and compared the results to those obtained with intact D-EVs. We found that the sonicated D-EVs had a much fainter inhibitory effect on biofilm growth than intact D-EVs (Fig. S4B), indicating that an intact EV structure is essential for extracellular vesicles to inhibit biofilm growth. These qualitative results are the first to demonstrate the potential of extracellular vesicles secreted by a bacterial biofilm in its survival/death phase for use against bacterial biofilm growth of the same bacterium.

**Fig. 3:**
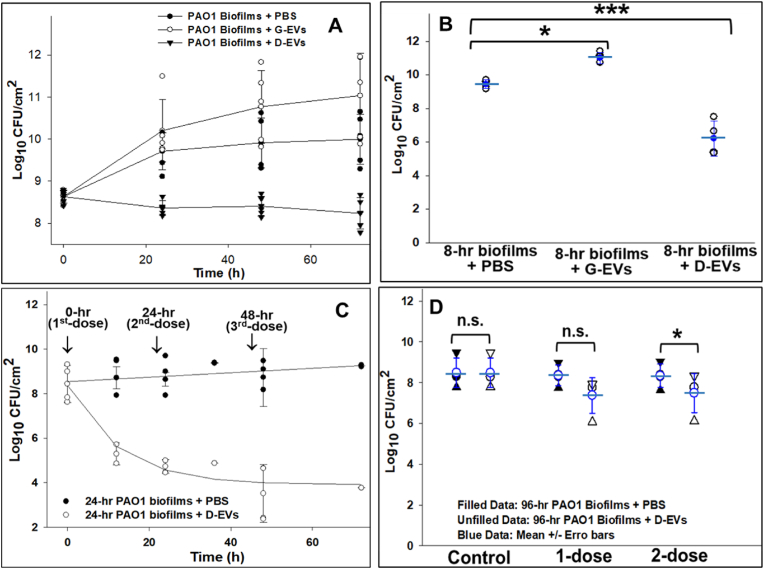
Functional effect of G-EVs and D-EVs on the growth behavior of *P. aeruginosa* PAO1 biofilms. **A**: Growth of *P. aeruginosa* PAO1 biofilms starting from the initial phase after one-dose treatment with G-EVs (0.028 μg/μL) and D-EVs (0.33 μg/μL), respectively. The data points are the times at which the biomass of the biofilms under each condition was collected and analyzed. **B**: Growth of *P. aeruginosa* PAO1 biofilms (8-h) formed during the log growth phase after one-dose treatment with G-EVs (0.037 μg/μL) or D-EVs (0.33 μg/μL). Biomasses were collected and analyzed 24 h after the biofilms were treated. **C**: Growth of *P. aeruginosa* PAO1 biofilms (24-h) developed during the stationary plateau phase after multidose treatment with D-EVs (0.33 μg/μL). Three doses of D-EVs were applied to the biofilms—at 0, 24 and 48 h (indicated by **↓** signs). The data points on each curve are the times at which the biomass of the biofilms under each condition was collected and analyzed. **D**: Growth of *P. aeruginosa* PAO1 biofilms (96-h) developed during the survival/death phase after one dose or 2 doses with a 12-h interval of D-EVs (0.33 μg/μL) or PBS. The mean value and error bar of each group of data are given in blue. For all data points, the biomasses of biofilms were collected and analyzed 24 h after each treatment. P value < 0.05 (*) and <0.01 (***).

To assess how D-EVs and G-EVs affect biofilm growth, we monitored *P. aeruginosa* PAO1 biofilm growth in terms of Log_10_ CFU/cm^2^. The functional efficacy of the extracellular vesicles varied depending on the maturity of the biofilm at each stage of development. Fig. 3A illustrates the growth of *P. aeruginosa* PAO1 bacterial biofilms grown for 2 h and treated with D-EVs and G-EVs compared to biofilms treated with PBS as a control. The results show that, compared to the control, the presence of G-EVs at a protein concentration of 0.028 μg/μL significantly increased the growth rate of the biofilms, while D-EVs at a protein concentration of 0.33 μg/μL showed a bactericidal effect on biofilm formation and growth. Fig. 3B shows the effects of D-EVs/G-EVs on the growth of *P. aeruginosa* PAO1 biofilms formed after 8 h of growth (the exponential growth phase) after bacterial inoculation. It reveals a ∼2-log_10_ increase in biofilm CFU counts after one dose of G-EVs at a protein content of 0.037 μg/μL. In contrast, treatment of the biofilm with one dose of D-EVs at a protein content of 0.33 μg/μL reduced CFU of biofilms ∼4.8-log_10_ compared to the control. These experiments demonstrated that one-dose treatment of D-EVs/G-EVs significantly affected the behavior of biofilms in the initial lag and exponential growth phases (2-h and 8-h biofilms). The results from the D-EV experiments suggest that D-EVs can be used to treat *P. aeruginosa* PAO1 biofilms at the early stage of biofilm growth. These quantitative results provide direct evidence supporting our hypothesis that G-EVs promote biofilm growth and D-EVs inhibit biofilm formation and growth.

Fig. 3C shows the inhibitory effects of multiple sequential doses of D-EVs at a protein concentration of 0.33 μg/μL on 24-h biofilms at the plateau stage. CFU analysis of the treated biofilms 24 h after the first dose showed a decrease in CFU of more than ∼3-log_10_ compared to the control. After further doses at 24 h and 48 h, respectively, biofilm growth was inhibited by an additional ∼1.5-log_10_. The observation of a total inhibition effect of 4.8-log_10_ suggests that a multidose approach using D-EVs could be used to treat mature biofilms.

After reaching the survival/death phase, biofilms live in a cryptic growth mode and secrete D-EVs to coordinate bacterial functions and maintain viable cells by gradually recycling nutrients derived from dead cells. In this mode, biofilms age, and they are more difficult to eradicate and more resistant to environmental stresses, including antibiotics [27,28]. We further investigated the effects of the extracted D-EVs on *P. aeruginosa* PAO1 biofilms (96-h) by applying either one dose of D-EVs at a protein concentration of 0.33 μg/μL directly to the aged biofilms or two doses separated by a 12-h interval. Analysis of the biomass collected 24 h after the D-EV treatments is shown in Fig. 3D; only <1-log_10_ reduction in *P. aeruginosa* PAO1 biofilms (96-h) was observed after 2 doses of D-EV treatment. The observed low inhibition effect was likely caused by the fact that the extracted D-EVs used for the treatment were secreted by *P. aeruginosa* PAO1 biofilms growing at the same developmental stage (after 96 h of growth).

### 2.3 Interactions between the extracellular vesicles and bacterial cells

**Fig. 4:**
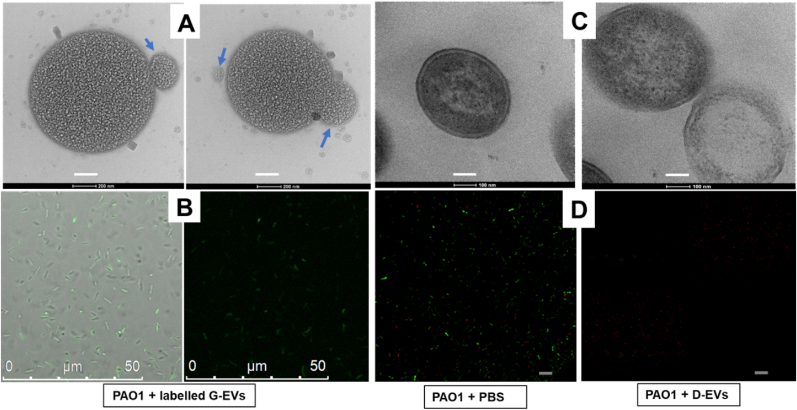
TEM and confocal laser scanning microscopy (CLSM) of the interactions of the G-EVs/D-EVs and *P. aeruginosa* PAO1 bacterial cells. **A**: TEM image of the bacterial cell after incubation with G-EVs added to the cell culture. After 24 h, the cells were negatively stained with 2% uranyl acetate before TEM images were taken (magnification power = 60000X and scale bar = 200 nm). Blue arrows indicate either an individual EV or an EV that is interacting with the *P. aeruginosa* cell. **B:** CLSM images of the bacterial cells that were incubated with DAPI-dye-labeled G-EVs. The images were taken with an excitation/emission of 359 nm/461 nm. Under this UV excitation, the recipient cells of DAPI-labeled G-EVs emit green fluorescence [78]. The image on the left shows an overlay of the brightfield and fluorescent images, while the image on the right shows the fluorescent image only. The green fluorescence indicates the presence of the DAPI-labeled G-EVs. **C**: TEM image of bacterial cells in the log phase after incubation with D-EVs. The cells were negatively stained with 2% uranyl acetate before TEM images were taken (magnification power = 40000X and scale bar = 100 nm). The image on the left shows a cell without D-EV treatment; double membrane layers are visible. The image on the right shows two cells treated with D-EVs. The membranes and cell walls of the cells have become damaged. The average sizes of *P. aeruginosa* PAO1 bacterial cells observed in images A and C are between 0.6 and 1.2 um, consistent with the literature report [79]. **D:** CLSM images of the bacterial cells that were positively stained with LIVE/DEAD BacLight Bacterial Viability Kits (excitation/emission: 480/500 nm for SYTO 9 and 490/635 nm for propidium iodide). The image on the left shows the green fluorescent live cells treated with PBS buffer; the image on the right shows the red dead cells (scale = 75 μm) after incubation with D-EVs. The total corrected cell fluorescence (TCCF) of control cultures and cultures treated with SYTO™ 9 was calculated using the ImageJ method (https://theolb.readthedocs.io/en/latest/imaging/measuring-cell-fluorescence-using-imagej.html):TCCF = Integrated Intensity – (Area of selected cell X Mean fluorescence of background readings).

To investigate whether the observed effects of D-EVs/G-EVs on *P. aeruginosa* PAO1 biofilm growth are due to direct interactions between the extracellular vesicles and target cells, we used TEM and CLSM to image potential interactions. The TEM images in Fig. 4A show that the G-EVs attached to the membrane surface of a target cell, triggering structural changes that led to EV uptake. In contrast, Fig. 4C shows that incubation with D-EVs led to deterioration of the bacterial cellular wall and subsequent cell death. These results are supported by 10.13039/501100007874CLSM experiments. Fig. 4B shows that *P. aeruginosa* PAO1 cells became green fluorescent after being incubated with DAPI-labeled G-EVs, indicating uptake of the DAPI-labeled G-EVs by the bacteria. To image bacterial cell viability, the bacterial cells were positively stained with LIVE/DEAD BacLight Bacterial Viability Kits. Fig. 4D shows that most bacterial cells in control samples (no D-EV presence) were alive and emitting green fluorescence with a total corrected cell fluorescence of 2.5 × 10^5^ ± 2.9 × 10^3^; however, after incubation with D-EVs, most of the cells were dead and emitting red fluorescence, indicating cell death; the green living cell total corrected fluorescence decreased to 3.1 × 10^3^ ± 1.4 × 10^2^. Overall, the finding that the G-EVs and D-EVs have different effects on bacterial cell structures may be directly related to the roles of G-EVs and D-EVs in promoting and inhibiting bacterial growth, respectively.

### 2.4 Proteomic analysis of the G-EVs and D-EVs

To further understand the different effects of D-EVs and G-EVs on PAO1 biofilm growth, proteomic profiles of the G-EVs and D-EVs were acquired. A total of 1099 and 987 proteins were found for G-EVs and D-EVs, respectively. Among these proteins, 79 surface and cytoplasmic proteins are shared by D-EVs and G-EVs with different abundances (Fig. S5). Additional proteomic profile analysis of G-EVs and D-EVs also identified a total of 92 (Table S1) and 77 (Table S2) highly abundant cytoplasmic proteins for the G-EV and D-EV samples, respectively. The protein profiles of these two populations of bacterial extracellular vesicles were found to be significantly different. The proteins carried by G-EVs were primarily associated with cell division and biosynthesis, DNA synthesis, and protein processes. The proteins carried by D-EVs, such as d-amino acid oxidase (DAO) domain-containing protein, d-amino acid dehydrogenase, quinone oxidoreductase, and 2,3-dihydro-3-hydroxy anthranilate isomerase, were mainly involved in inhibiting cell growth, reactive oxygen species (ROS) production, and iron acquisition to promote cell survival or induce cell death.

The analysis also identified some key surface proteins that were uniquely present in different abundances on the surfaces of the D-EVs and G-EVs, which may contribute to the unique roles of D-EVs and G-EVs in biofilm growth. These proteins are listed in Table 1. Among them, two proteins (**#1-#2**) were exclusively present on the G-EV surface, while 13 (**#3**-**#15**) were exclusively present on the D-EV surface. Protein **#1** on the G-EV surface is involved in the sulfate transport system of bacteria, which is responsible for energy coupling to the transport system, while protein **#2** belongs to the CusCFBA copper efflux system, which plays a crucial role in copper homeostasis within the bacteria [29]. With these important functional proteins on their surface, the G-EVs may enhance bacterial viability and promote cell growth under normal culture conditions through interactions with target bacterial cells.

**Table 1:** Profiles of key surface proteins on G-EVs and D-EVs from PAO1 biofilms.

No.	Proteins	Possible functions	G-EV	D-EV
**1**	Sulfate/thiosulfate import ATP-binding protein CysA	The sulfur-regulated gene (CysA) that encodes the membrane-associated ATP-binding protein of the sulfate transport system of bacteria is responsible for energy coupling to the transport system.	**H**	**x**
**2**	HlyD_D23 domain-containing protein	The membrane fusion proteins of the CusCFBA copper efflux system that play a crucial role in copper homeostasis within the bacteria, which is directly associated with bacterial viability.	**H**	**x**
**3**	Heme/hemoglobin uptake outer membrane receptor PhuR	Promote acquisition of heme as iron resource for bacterial growth. Because of its capability of transferring electrons at physiological pH, iron plays a critical role in bacterial physiology as an essential component of metabolic enzymes and regulatory proteins.	**x**	**H**
**4**	Hemin receptor		**x**	**H**
**5**	Putative outer membrane ferric siderophore receptor		**x**	**H**
**6**	Putative hydroxamate-type ferrisiderophore receptor		**x**	**H**
**7**	Heme acquisition protein HasAp		**x**	**H**
**8**	Ferripyoverdine receptor		**x**	**H**
**9**	Extracellular heme-binding protein		**x**	**H**
**10**	TonB-dependent receptor	Either mediates the release of iron into the periplasm of the bacteria or transports ferric enterobactin into the periplasm.	**x**	**H**
**11**	Putative TonB-dependent receptor		**x**	**H**
**12**	Ferric enterobactin receptor		**x**	**H**
**13**	AMP-binding protein	Regulatory enzyme in response to environmental changes.	**x**	**H**
**14**	Quinone oxidoreductase-like protein 2	Reduces quinones into hydroquinone, without proton translocation.	**x**	**H**
**15**	2,3-dihydro-3-hydroxyanthranilate isomerase	Phenazine-1-carboxylate biosynthesis/important for redox cycling.	**x**	**H**

**Note:** H – high abundancy; x – not found. n = 3.

The thirteen D-EV surface proteins can be categorized into three groups. The first group of D-EV surface proteins (**#3-#9)** are membrane receptors or functional proteins that enhancing the acquisition of iron resources. The second group (**#10-#12**) includes membrane receptors that transport ferric enterobactin into the periplasm or release iron into the periplasm of the bacteria. Iron is an essential component of metabolic enzymes and regulatory proteins that support the growth and survival of most bacterial species. However, excessive uptake of iron by bacteria can be detrimental because of the iron-triggered Fenton/Haber-Weiss reaction, which produces harmful ROS such as superoxide (O^2−^), hydrogen peroxide (H_2_O_2_), and the highly destructive hydroxyl radical (˙OH). Accumulation of ROS can inactivate key functional enzymes and activate bacterial programmed cell death [[30], [31], [32], [33], [34], [35], [36], [37]]. The last group of D-EV surface proteins (**#14-#15**) includes quinone oxidoreductase and 2,3-dihydro-3-hydroxyanthranilate isomerase, which participate directly in the Fenton reaction and ROS generation.

### 2.5 Mechanistic investigations of inhibition effects of the D-EVs on biofilm growth

**Fig. 5:**
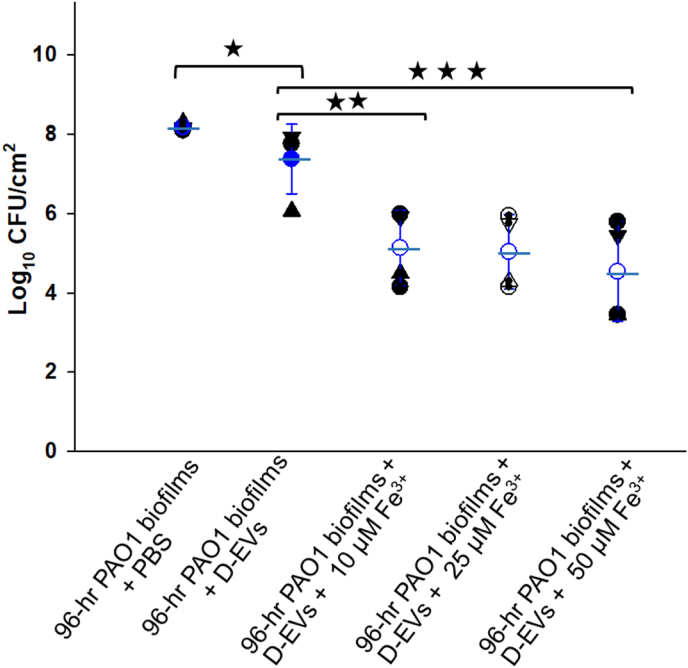
Synergic effects of D-EVs/Fe^3+^ on 96-h *P. aeruginosa* PAO1 biofilm growth. For all data points, biomasses of biofilms were collected and analyzed 24 h after each treatment. The mean value and error bar of each group of data are given in blue. P-value <0.05 (*), <0.03 (**) and <0.01(***).

Based on the information about these unique ferric transportation/acquisition-related proteins observed on D-EV surface, we hypothesized that D-EVs induce excessive iron uptake by the D-EV recipient bacterial cells, resulting in the accumulation of ROS and ultimately the activation of bacterial cell death. This hypothesis was tested by examining whether the efficacy of the D-EVs against aged biofilms grown for 96 h (Fig. 3D) could be improved in the presence of ferric ions. Compared to the PBS control, the presence of ferric ions alone marginally promoted biofilm growth, from ∼8.2-log_10_ to 8.7-log_10_ (Fig. S6B), while 2-dose treatments of D-EV alone only reduced the aged biofilm growth by less than ∼1-log_10_ (Fig. S6A). However, when ferric ions were applied to the 96-h *P. aeruginosa* PAO1 biofilms together with D-EVs, the growth of the biofilms was significantly inhibited (Fig. 5), and the inhibition efficiency was found to be Fe^3+^ concentration dependent. As [Fe^3+^] increased from 10 μM to 50 μM, the inhibition of biofilm growth increased from a ∼2.6-log_10_ reduction to a ∼3.4-log_10_ reduction. These results demonstrate that both D-EVs and ferric ions are needed to improve biofilm treatment efficiency.

**Fig. 6:**
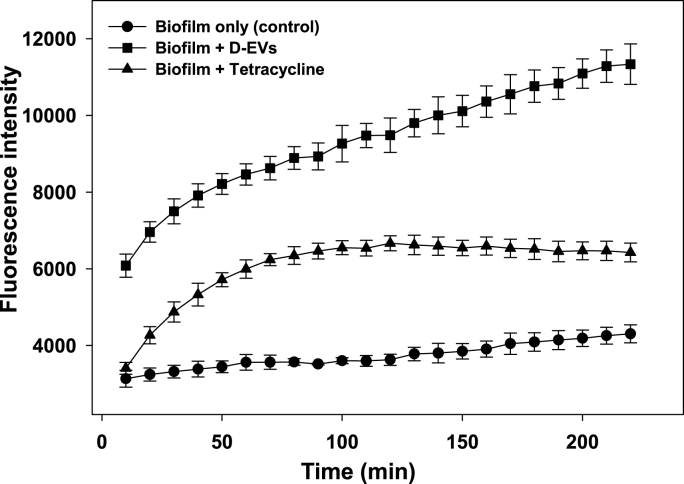
ROS levels of *P. aeruginosa* PAO1 biofilms in the lag phase were monitored under various conditions. After 1 h of incubation with dye agents from a Cellular ROS Assay Kit (Red) (ab186027), *P. aeruginosa* PAO1 bacteria in the lag phase were mixed with PBS buffer, tetracycline (10 mg/ml), or D-EVs (97.9 μg/ml), immediately followed by monitoring of changes in their fluorescence intensity (excitation/emission: 520/605 nm). An increase in fluorescence intensity was correlated with an increase in overall ROS levels in each sample.

Using a commercial cellular ROS assay kit, we observed that the biofilms treated with D-EVs produced much higher levels of ROS than the negative control biofilm (treated with PBS buffer) and the positive control biofilm (treated with tetracycline at 10 mg/mL) (Fig. 6). Although specific ROS species were not identified in this measurement, these results provide evidence that D-EVs promote iron uptake and induce overall ROS accumulation within the biofilm, ultimately leading to cell death.

To determine the potential cytotoxicity of *P. aeruginosa* PAO1 D-EVs to mammalian cellular function, we examined the viability of human mesenchymal stem cells (hMSC) in the presence of the D-EVs (0.33 μg/μL) (see Supplementary Information section S5). The results showed no significant detrimental effects on either cell growth (Fig. S7A) or cellular morphology (Fig. S7B) of hMSC after exposure to the D-EVs for two days. These observations are consistent with literature studies on the biosafety of using bacterial extracellular vesicles in human bone and tissue therapy [38].

## 3 Discussion

Bacterial biofilms are complex surface-attached communities of bacteria held together by self-produced polymer matrices. The formation of biofilms is a complex process and involves multiple stages. As they enter a different growth stage, bacteria quickly respond to changes in the environment to propagate or survive by rapidly communicating with neighboring cells and reorganizing their intracellular physiological processes. Bacterial extracellular vesicles are believed to be one of the key players in bacterial intercellular communications.

*P. aeruginosa* is an antibiotic-resistant Gram-negative organism and has been extensively studied for the production and characterization of extracellular vesicles secreted by planktonic cells or biofilms [[39], [40], [41]]. These studies provide a basis for us to understand the different pathways of EV secretions [42,43], the role of EVs in trafficking cellular signals such as quorum sensing signals to facilitate cellular activities in planktonic cells and biofilms [18,44], the biogenesis of biofilm EVs [43], and proteome profiles of the extracellular vesicles and extracellular matrix of *P. aeruginosa* biofilms [43,[45], [46], [47], [48]]. Despite this extensive reported research, an understanding of the comprehensive roles of EVs in controlling *P. aeruginosa* biofilm growth is still elusive because the vesicle components and functional roles strongly depend on the conditions of the biofilm growth stage and EV secretions [43,49].

In this study, we report that extracellular vesicles secreted by *P. aeruginosa* PAO1 biofilms at different growth stages can have different effects on the same population of *P. aeruginosa* PAO1 biofilms, meaning that the functional effects of extracellular vesicles are conditional, or environment-dependent. For example, the growth of 2-h *P. aeruginosa* PAO1 biofilms can be enhanced by ∼2-log_10_ CFU/cm^2^ in the presence of G-EVs; however, the growth can be inhibited by the presence of their D-EVs (Fig. 2A). Our results also show that the presence of the D-EVs eliminated 8-h and 24-h *P. aeruginosa* PAO1 biofilms (Fig. 2B and C). G-EVs and D-EVs are both secreted by *P. aeruginosa* PAO1 biofilms but at different biofilm growth stages. G-EVs are secreted in the exponential growth phase, while D-EVs are secreted in the survival/death phase. These results suggest that the functional roles of extracellular vesicles are closely correlated with the functions of the parental cells under the specific conditions under which the extracellular vesicles are secreted.

Because of the rich nutrition and favorable growth conditions of the exponential growth phase, bacteria optimize their internal cellular processes to expand their population rapidly and establish biofilm communities by activating bacterial attachments [50]. During this active growth period, bacteria likely secrete G-EVs as messengers to coordinate the cellular actions of neighbors to establish biofilm communities. Our analysis of the proteomic profiles of G-EVs suggests that most of the abundant proteins/enzymes found in G-EVs are those involved in cellular processes for growth, DNA synthesis, and protein synthesis/processes (Table 1 and Table S1). As key cellular communication mediators, EVs are known to assist in transferring DNA, RNA, proteins, and other molecules to target cells [40,51]. Because G-EVs enrich proteins that are essential for cellular growth, once they are taken up by other bacterial cells, these proteins can likely enhance the bacterial functions associated with these proteins, such as cellular attachment, and promote biofilm development and cell growth.

Death and growth rates are balanced once biofilm enters the stationary phase while biofilm matures. At this stage, the bacteria within the biofilm become more resistant to antibiotics [52]. Under normal culture conditions or in favorable growth environments, mature biofilms ultimately enter the dispersal/detachment phase, during which bacterial cells depart from the biofilm to start or participate in a new biofilm formation cycle. However, when nutrients are depleted, cells starve. This affects the biofilm in two ways. First, some bacterial cells within the biofilm die to generate nutrients by decreasing the population density (increasing the amount of nutrients per cell) and releasing nutrients through dead cells to provide nutrition for the remaining nutrient-deprived cell population [33,[53], [54], [55], [56], [57]]. Second, to survive in such a stressful condition, the core group of biofilm bacteria rapidly adjusts its intracellular metabolic pathways, leading to alterations in intracellular components and surface proteins. Meanwhile, extracellular vesicles (D-EVs) are secreted by the core bacteria to reflect these alterations. The secreted D-EVs then serve as cellular messengers to coordinate the actions of the core population of bacteria and enhance their ability to acquire vital supplies and nutrition from a resource-scarce environment.

Several unique proteins were identified on the surface of the D-EVs (Table 1), all belonging to a group of proteins that help bacterial growth by acquiring iron resources from nutrition-depleted environments. Iron is an essential component of metabolic enzymes and regulatory proteins; it acts as an electron carrier and a key nutrient for bacterial life and thus plays a critical role in bacterial physiology to support growth. Therefore, it is likely that D-EVs serve as unique messengers and protein carriers to help bacterial survival under conditions of nutrition/iron deprivation by coordinating bacterial cellular functions and delivering key proteins to the recipient cells to enhance their nutrition acquisitions. However, D-EVs can only benefit recipient bacterial cell growth/survival under nutrition depletion conditions. In normal or iron-rich culture environments, D-EVs can be toxic to bacteria. For instance, as shown in Fig. 2, the presence of D-EVs can effectively inhibit the growth of parental *P. aeruginosa* PAO1 biofilms under normal culture conditions. Furthermore, the inhibition power of D-EVs against 96-h *P. aeruginosa* PAO1 parental biofilms can be significantly enhanced by the presence of extra Fe^3+^ (Fig. 4).

Maintaining an intracellular iron concentration of 10^−6^ M is essential for bacterial growth; therefore, iron uptake is strictly regulated by bacterial cellular processes [30,58]. Insufficient iron uptake can lead directly to cell starvation, while excessive iron uptake in a medium-rich environment can result in bacterial cell death by activating ferroptosis, an iron-dependent form of programmed cell death [59,60]. A consequence of ferroptosis activated by a high intracellular concentration of iron is the triggering of the Fenton/Haber-Weiss reaction, which produces lethal concentrations of ROS, leading to cell death [30]. This is supported by the results shown in Fig. 5, which demonstrate that *P. aeruginosa* PAO1 bacteria produced more overall ROS when exposed to D-EVs than when exposed to an antibiotic drug.

In addition to the unique surface proteins associated with bacterial iron acquisition (Table 1), analysis of the proteomic profile of D-EVs identified a number of unique cytoplasmic proteins that are exclusive to D-EVs. These proteins include d-amino acid oxidase (DAO) domain-containing protein, d-amino acid dehydrogenase, quinone oxidoreductase, and 2,3-dihydro-3-hydroxy anthranilate isomerase (Table S2). They may play important roles in activating ferric-based programmed bacterial cell death. For example, d-amino acid oxidase (DAO) domain-containing protein and d-amino acid dehydrogenase are involved in catalyzing the oxidative deamination of d-amino acids to produce hydrogen peroxide (H_2_O_2_) in bacteria [61], which can then participate in the Fenton reaction to generate ROS species of hydroxy radical (HO^•^) in the presence of ferric ions. Quinone oxidoreductase and 2,3-dihydro-3-hydroxy anthranilate isomerase are known to be involved in a pathway converting quinone to hydroquinone and participate in the redox cycle of phenazine/pyocyanin to produce ROS species of superoxide ions (O_2_^−^) [62,63]. The proteins carried by D-EVs provide support to the mechanism underlying the observed inhibition effects of D-EVs on biofilm growth; i.e., after being taken up by *P. aeruginosa* PAO1 cells in the presence of excess Fe^3+^ ions, the D-EVs can activate ferric-based programmed cell death and trigger ROS production by the recipient cells, thus inhibiting bacterial biofilm growth.

In summary, our study provides strong evidence of the importance of bacterial extracellular vesicles in regulating biofilm growth. The regulatory roles of bacterial extracellular vesicles depend on the biofilm growth stage at which the extracellular vesicles are secreted. In addition, we discovered dual roles of D-EV in controlling biofilm growth: 1) help bacterial survival under nutrition depletion conditions and 2) treat *P. aeruginosa* PAO1 biofilms in the presence of Fe^3+^ ions. Our discovery brings both excitement and challenge to the field. The excitement is that the discoveries, especially the Fe^3+^-enhanced inhibition power of D-EVs, provides an alternative for developing new therapies against drug-resistant bacterial infections. The challenge is that our results raise many fundamental questions. For example, how do environmental signals/conditions at each stage of bacterial biofilm development affect EV biogenesis? Do extracellular vesicles secreted by other types of bacteria have the same properties and use a similar mechanism to inhibit biofilm growth? Are there other mechanisms involved? What are the roles of extracellular vesicle DNA/RNA components in the mechanisms, and how do they change when extracellular vesicles are secreted by bacteria at different developmental stages? How do intracellular pathways of biomass change in response to exposure to D-EV? Answers to these questions will shed light on the regulatory roles and detailed mechanisms of bacterial extracellular vesicles in biofilm growth and how the roles switch from one stage to another. Furthermore, our data suggest that the inhibition of biofilm growth by D-EVs involves ROS production. However, the nature of the ROS species involved in the inhibition is unknown. Answering this question will help us pinpoint the intracellular mechanism of D-EV-induced cell death. Also, is it possible that the observed condition-dependent regulatory roles of bacterial extracellular vesicles can be applied to other cellular systems? Answering this question may help address the challenges faced in the clinical application of mammalian or stem-cell-derived extracellular vesicle-based therapies for various disease treatments [[64], [65], [66]].

## 4 Materials and methods

### 4.1 Biofilms

*P. aeruginosa* (PAO1) [67] from a −80 °C freezer was streaked on tryptic soy agar (TSA, BD Difco™ Dehydrated Culture Media, DF0369-17-6) and incubated overnight at 37 °C. The *P. aeruginosa* was maintained on TSA plates at 4 °C and an inoculum was prepared by transferring a touch from this plate into 10 mL of tryptic soy broth (BD Bacto™) and incubated overnight. Then, a volume of 100 μL (OD_600_ = 0.1) was used to seed 47-mm sterilized filter membranes (Membrane Solutions MCE Gridded Membrane Filter, Mixed Cellulose Esters Membrane Filter, Pore Size: 0.45 μm) on TSA petri dishes. The plates were incubated at either 37 °C or 33 °C. At desired time points (0, 1, 2, 4, 8, 12, 16, 24, 32, 48, 72, 85, 96, and 120 h), the biofilm was suspended in 0.9% NaCl to prepare serial dilutions for colony forming unit (CFU) counting. The viable cell concentration (CFU/cm^2^) was calculated according to the literature [68,69]. All experiments were done under aseptic conditions. TSA and TSB were autoclaved at 121 °C and 1.5 psa for 50 min/L.

### 4.2 Extracellular vesicle extraction

Biofilms for extracellular vesicle extraction were grown at 33 °C on 47-mm sterilized membrane paper (Membrane Solutions MCE Gridded Membrane Filter, Mixed Cellulose Esters Membrane Filter, Pore Size: 0.45 μm) on TSA Petri dishes for 8 h or 96 h. Extracellular vesicles were isolated and purified from *P. aeruginosa* PAO1 biofilms using a conventional differential centrifugation protocol [[21], [22], [23], [24]]. Briefly, multiple 47-mm sterilized membrane papers were inoculated with 4.4 × 10^5^ ± 7.3 × 10^2^ CFU/cm^2^ on TSA plates and incubated. When biofilms reached their exponential growth phase, their extracellular vesicles were extracted and defined as growth extracellular vesicles (G-EVs). Similarly, when biofilms reached their death/survival growth phase, their extracellular vesicles were extracted and defined as death extracellular vesicles (D-EVs). These processes are illustrated in Fig. 2. For extracellular vesicle extraction, the biofilms were removed from the membrane and then suspended in TSB. The cell debris was removed by centrifugation at 10,000 g for 30 min at 4 °C using a benchtop centrifuge (Biofuge, HERAEUS). Then, the supernatant was collected and filtered using a 0.45-μm syringe filter (GenClone 25–246, Syringe Filters, PES, 30-mm Diameter, Sterile, Cat #: 25–246). The filtrate was ultracentrifuged (Optima LE-80K Ultracentrifuge, Beckman) at 150,000 g for 2 h at 4 °C, twice, and filtered with a 0.22-μm filter (GenClone 25–244, Syringe Filters, PES, 30-mm Diameter, Sterile, Cat #: 25–244) after the first run to remove macrovesicles >200 μm in size. PBS was used as a buffer to suspend the pellets after each ultracentrifugation run. The extracted extracellular vesicles were stored at −20 °C for further analysis or use.

### 4.3 Testing activity of extracellular vesicles against biofilm at different ages

To investigate the functional effect of extracted extracellular vesicles on the growth of *P. aeruginosa* PAO1 biofilms, we used the Kirby-Bauer disk diffusion susceptibility test [70,71]. Briefly, an inoculum was prepared by activating a touch from the *P. aeruginosa* PAO1-TSA plates in TSB overnight. Then, the bacterial suspension was adjusted to OD_600_ ∼0.1, and the adjusted suspension was spread onto TSA plates using 6-inch sterilized cotton swabs and left for 2 h. Discs were loaded with 25 μL of PBS; tetracycline (Cat # 87128, Sigma-Aldrich) at 0.033 μg/μL, 0.22 μg/μL, and 1 μg/μL; G-EVs at 0.037 μg/μL; and D-EVs at 0.11 μg/μL, 0.22 μg/μL, and 0.33 μg/μL, respectively. The treated discs were placed on the bacteria-seeded TSA plates and incubated at 37 °C overnight.

To quantify the effects of G-EVs and D-EVs on the growth behavior of *P. aeruginosa* PAO1 biofilms, biofilms grown for 2 h (lag phase), 8 h (exponential phase), and 24 h (stationary phase) were tested. Briefly, an inoculum was prepared by transferring a touch from the *P. aeruginosa* PAO1-TSA plates into 10 ml of TSB and incubating the plates at 37 °C overnight. A volume of 2.5 μL (OD_600_ ∼0.5) was used to seed sterile 13-mm polycarbonate membranes (WHA10417401, Sigma‐Aldrich) on TSA plates and incubated at 37 °C. Biofilms grown for 2 h were treated with one dose of G-EVs (0.028 μg/μL) or D-EVs (0.33 μg/μL) and incubated at 37 °C for 24 h. Biofilms grown for 8 h were treated with one dose of G-EVs (0.037 μg/μL) or D-EVs (0.33 μg/μL) and incubated at 37 °C for 24 h. Three doses of D-EVs (0.33 μg/μL) were applied (one dose every 24 h) to biofilms grown for 24 h, and the plates were incubated at 37 °C. Then viable cells were counted and reported as CFU/cm^2^.

### 4.4 Bacterial cells, biofilms and EV imaging

We used a Leica SP-5 confocal laser scanning microscope to image biofilms grown for 8 h. The biofilms, planktonic cells and controls were stained with LIVE/DEAD BacLight Bacterial Viability Kits following published literature [72]. Excitations/emissions of 480/500 nm for SYTO 9 and 490/635 nm for propidium iodide were used.

Transmission electron microscopy (TEM, FEI Tecnai G2 20 Twin equipped with a 200-KV LaB6 electron source) was used to image single cells and extracellular vesicles. *P. aeruginosa* PAO1 biofilms grown for 8 h were incubated with D-EVs for 24 h. Cells were suspended in PBS for 5 min; then, cells were collected, followed by adding PBS and osmium at 3:1 (v:v). The mixture was then incubated at 4 °C overnight. After the resin was removed, the samples were washed with distilled water and exposed to a series of ethanol concentrations (30%, 50%, 70%, 90%, and 100%). Subsequently, the cells were mixed with Spurr in propylene oxide and left on the shaker overnight. The next day, 100% Spurr was added, and the samples were kept at 60 °C for 24 h. Blocks were trimmed, and 70-nm thin sections were prepared and loaded onto formvar/carbon-coated copper EM grids with a thickness of 200 nm. The grids were positively stained with 2% uranyl acetate and lead. Grids were examined using TEM.

The direct interactions between G-EVs and bacterial cells were investigated using TEM and CLSM. To investigate using TEM, 8-h *P. aeruginosa* PAO1 biofilms were incubated with G-EVs for 24 h. Four μL of the cell sample were loaded onto formvar/carbon-coated copper EM grids with a thickness of 200 nm. The grids were then stained with 4 μL of 2% uranyl acetate and examined using TEM. For CLSM, 2-h *P. aeruginosa* PAO1 biofilms were incubated with 10 μL of DAPI-dye-labeled G-EVs at 37 °C in the dark for 24 h. DAPI (diamidino-2-phenylindole) stains extracellular vesicle DNA. Five μL of the treated cell suspension were loaded onto a glass slide and examined with CLSM using an excitation/emission wavelength of 359/461 nm.

### 4.5 Qualitative fluorescence-based assay of extracellular vesicles

Extracellular vesicles were labeled with Vybrant™ DiI Cell-Labeling Solution according to the manufacturer's protocol. To prepare the staining medium, 5 μL of the labeling solution is added to 1 mL of PBS. The extracellular vesicles are then incubated with the staining medium at 37 °C for 20 min. After incubation, a small volume (5 μL) of the stained suspension is placed on a glass slide and examined under a confocal laser scanning microscope (Leica SP-5 Confocal Laser Scanning Microscope). The excitation and emission wavelengths used for imaging are 549 and 565 nm, respectively. This method can provide valuable insights into the cellular uptake and distribution of extracellular vesicles labeled with the fluorescent dye [[73], [74], [75]].

### 4.6 Quantitative protein-based analysis of extracellular vesicles

To quantify the protein concentration of the extracted extracellular vesicle samples, a modified bicinchoninic acid assay (BCA) protocol was used (Pierce™ BCA Protein Assay Kit, Thermo Fisher Scientific) [76]. The protocol involved denaturing equal amounts of extracted extracellular vesicles (25 μL) using RIPA 5x for 30 min on ice, followed by protein precipitation after the addition of trichloroacetic acid (TCA, Sigma-Aldrich, 91228), (v:v, 1:0.25 of sample: TCA). The resulting precipitate was collected by centrifugation at 6000 rpm for 5 min and washed with cold acetone. The precipitate was then dissolved in PBS (25 μL) and mixed with an equal amount of the working solution (50 parts reagent A, 48 parts reagent B, and 2 parts reagent C) in a 96-well plate. The plate was incubated at 37 °C for 2 h, and the absorbance was measured at 562 nm using a Cytation^TM^ 5 plate reader.

### 4.7 Proteomic analysis

Proteomic identifications of extracted G-EVs and D-EVs were done using Proteome Discoverer (Thermo Orbitrap Fusion Tribrid). A 100-μL volume of extracellular vesicles was centrifuged for 1 h at 4 °C and 150,000×*g*. The pellet was dissolved in 50 μL of 6 M guanidine hydrochloride (GuHCL, Thermo Scientific™, AAJ6078622) and placed on the shaker for 20 min at 1000 rpm; then, samples were sonicated 4 times for 10 s with a 2-min ice cooling interval and stored at −70 °C.

### 4.8 Reactive oxygen species detection

We used a Cellular ROS Assay Kit (Red) (ab186027) to measure ROS in our biofilm samples following the modified manufacturer's protocol (Abcam). Briefly, A 25-μL (OD_600_ = 0.1) sample was transferred to each well in a 96-well plate and incubated for 7 h. A 100-μL volume of ROS working solution was added to each well and incubated for 1 h, followed by the addition of test samples (D-EVs (0.098 μg/μL), tetracycline (10 mg/ml), or PBS). The plate was placed in the plate reader for immediate monitoring of the change in ROS levels by measuring the fluorescence increase at excitation/emission 520/605 nm (cutoff 590 nm). The ROS working solution was prepared by mixing 40 μL of DMSO with the red stain; then, 5 μL of the mixture was mixed with 2.5 ml of buffer.

### 4.9 Statistical analysis

All experiments were done in biological replicates, and the number of biological replicates is given for each experiment. The data were averaged and are presented as the standard error of the mean. The statistical significance of the differences between treatments was determined using the Wilcoxon rank sum test. A p-value less than 0.05 was considered significant [77].

## Competition interests statement

All authors declare no conflicts of interest.

## CRediT authorship contribution statement

**Marwa Gamal Saad:** Conceptualization, Formal analysis, Investigation, Writing – original draft. **Haluk Beyenal:** Funding acquisition, Supervision, Writing – review & editing. **Wen-Ji Dong:** Conceptualization, Funding acquisition, Project administration, Resources, Supervision, Writing – review & editing.

## Data Availability

Data will be made available on request.

## References

[1] Clinic, M. Drugs and supplements: tetracycline (class) (oral route, parenteral route)..

[2] De Oliveira D.M.P. (2020). Antimicrobial resistance in ESKAPE pathogens. Clin Microbiol Rev.

[3] de Kraker M.E.A., Stewardson A.J., Harbarth S. (2016). Will 10 million people die a year due to antimicrobial resistance by 2050?. PLoS Med.

[4] Davies D. (2003). Understanding biofilm resistance to antibacterial agents. Nat Rev Drug Discov.

[5] Mah T.F. (2012). Biofilm-specific antibiotic resistance. Future Microbiol.

[6] Aertsen A., Michiels C.W. (2004). Stress and how bacteria cope with death and survival. Crit Rev Microbiol.

[7] Kim D.K. (2013). EVpedia: an integrated database of high-throughput data for systemic analyses of extracellular vesicles. J Extracell Vesicles.

[8] Bitto N.J., Kaparakis-Liaskos M. (2017). The therapeutic benefit of bacterial membrane vesicles. Int J Mol Sci.

[9] Grande R. (2015). Helicobacter pylori ATCC 43629/NCTC 11639 outer membrane vesicles (OMVs) from biofilm and planktonic phase associated with extracellular DNA (eDNA). Front Microbiol.

[10] Bebelman M.P. (2018). Biogenesis and function of extracellular vesicles in cancer. Pharmacol Therapeut.

[11] Colombo M., Raposo G., Thery C. (2014). Biogenesis, secretion, and intercellular interactions of exosomes and other extracellular vesicles. Annu Rev Cell Dev Biol.

[12] Raposo G., Stoorvogel W. (2013). Extracellular vesicles: exosomes, microvesicles, and friends. JCB (J Cell Biol).

[13] Riches A. (2014). Regulation of exosome release from mammary epithelial and breast cancer cells - a new regulatory pathway. Eur J Cancer.

[14] Silva J. (2012). Analysis of exosome release and its prognostic value in human colorectal cancer. Genes Chromosomes Cancer.

[15] Suetsugu A. (2013). Imaging exosome transfer from breast cancer cells to stroma at metastatic sites in orthotopic nude-mouse models. Adv Drug Deliv Rev.

[16] Molina-Tijeras J.A., Galvez J., Rodriguez-Cabezas M.E. (2019). The immunomodulatory properties of extracellular vesicles derived from probiotics: a novel approach for the management of gastrointestinal diseases. Nutrients.

[17] Wolf T., Baier S.R., Zempleni J. (2015). The intestinal transport of bovine milk exosomes is mediated by endocytosis in human colon carcinoma caco-2 cells and rat small intestinal IEC-6 cells. J Nutr.

[18] Mashburn L.M., Whiteley M. (2005). Membrane vesicles traffic signals and facilitate group activities in a prokaryote. Nature.

[19] Schooling S.R., Hubley A., Beveridge T.J. (2009). Interactions of DNA with biofilm-derived membrane vesicles. J Bacteriol.

[20] Mashburn-Warren L.M., Whiteley M. (2006). Special delivery: vesicle trafficking in prokaryotes. Mol Microbiol.

[21] Romancino D.P. (2013). Identification and characterization of the nano-sized vesicles released by muscle cells. FEBS Lett.

[22] Alvarez M.L. (2012). Comparison of protein, microRNA, and mRNA yields using different methods of urinary exosome isolation for the discovery of kidney disease biomarkers. Kidney Int.

[23] Liga A. (2015). Exosome isolation: a microfluidic road-map. Lab Chip.

[24] Rekker K. (2014). Comparison of serum exosome isolation methods for microRNA profiling. Clin Biochem.

[25] Willms E. (2018). Extracellular vesicle heterogeneity: subpopulations, isolation techniques, and diverse functions in cancer progression. Front Immunol.

[26] Mathieu M. (2021). Specificities of exosome versus small ectosome secretion revealed by live intracellular tracking of CD63 and CD9. Nat Commun.

[27] Schink S. (2022). Analysis of proteome adaptation reveals a key role of the bacterial envelope in starvation survival. Mol Syst Biol.

[28] Watson S.P., Clements M.O., Foster S.J. (1998). Characterization of the starvation-survival response of Staphylococcus aureus. J Bacteriol.

[29] Meir A. (2019). Inhibiting the copper efflux system in microbes as a novel approach for developing antibiotics. PLoS One.

[30] Cornelis P. (2011). Iron homeostasis and management of oxidative stress response in bacteria. Metallomics.

[31] Yeom J., Imlay J.A., Park W. (2010). Iron homeostasis affects antibiotic-mediated cell death in Pseudomonas species. J Biol Chem.

[32] Hong Y. (2019). Post-stress bacterial cell death mediated by reactive oxygen species. Proc Natl Acad Sci U S A.

[33] Peeters S.H., de Jonge M.I. (2018). For the greater good: programmed cell death in bacterial communities. Microbiol Res.

[34] Allocati N. (2015). Die for the community: an overview of programmed cell death in bacteria. Cell Death Dis.

[35] Eid R., Arab N.T., Greenwood M.T. (2017). Iron mediated toxicity and programmed cell death: a review and a re-examination of existing paradigms. Biochim Biophys Acta Mol Cell Res.

[36] Tanouchi Y. (2013). Programmed cell death in bacteria and implications for antibiotic therapy. Trends Microbiol.

[37] Ramisetty B.C.M., Sudhakari P.A. (2020). Bacterial Programmed Cell Death': cellular altruism or genetic selfism?. FEMS Microbiol Lett.

[38] Liu H. (2022). Bacterial extracellular vesicles-based therapeutic strategies for bone and soft tissue tumors therapy. Theranostics.

[39] Tashiro Y., Uchiyama H., Nomura N. (2012). Multifunctional membrane vesicles in Pseudomonas aeruginosa. Environ Microbiol.

[40] Henriquez T., Falciani C. (2023). Extracellular vesicles of Pseudomonas: friends and foes. Antibiotics (Basel).

[41] Kulp A., Kuehn M.J. (2010). Biological functions and biogenesis of secreted bacterial outer membrane vesicles. Annu Rev Microbiol.

[42] Toyofuku M. (2019). Bacterial communication through membrane vesicles. Biosci Biotechnol Biochem.

[43] Cooke A.C. (2019). Analysis of Pseudomonas aeruginosa biofilm membrane vesicles supports multiple mechanisms of biogenesis. PLoS One.

[44] Diggle S.P. (2007). The Pseudomonas aeruginosa 4-quinolone signal molecules HHQ and PQS play multifunctional roles in quorum sensing and iron entrapment. Chem Biol.

[45] Couto N. (2015). Proteome profiles of outer membrane vesicles and extracellular matrix of biofilms. J Proteome Res.

[46] Zavan L. (2023). The mechanism of outer membrane vesicle biogenesis determines their protein composition. Proteomics.

[47] McMillan H.M., Kuehn M.J. (2023). Proteomic profiling reveals distinct bacterial extracellular vesicle subpopulations with possibly unique functionality. Appl Environ Microbiol.

[48] Hou M. (2023). Deep profiling of the proteome dynamics of Pseudomonas aeruginosa reference strain PAO1 under different growth conditions. J Proteome Res.

[49] Juodeikis R., Carding S.R. (2022). Outer membrane vesicles: biogenesis, functions, and issues. Microbiol Mol Biol Rev.

[50] Gilbert P. (2002). The physiology and collective recalcitrance of microbial biofilm communities. Adv Microb Physiol.

[51] Toyofuku M. (2019). Bacterial communication through membrane vesicles. Biosc Biotech Biochem.

[52] Singh S. (2017). Understanding the mechanism of bacterial biofilms resistance to antimicrobial agents. Open Microbiol J.

[53] Monds R.D., O'Toole G.A. (2009). The developmental model of microbial biofilms: ten years of a paradigm up for review. Trends Microbiol.

[54] Bayles K.W. (2014). Bacterial programmed cell death: making sense of a paradox. Nat Rev Microbiol.

[55] Corchero J.L. (2001). Cell lysis in Escherichia coli cultures stimulates growth and biosynthesis of recombinant proteins in surviving cells. Microbiol Res.

[56] Takano S. (2017). Density-dependent recycling promotes the long-term survival of bacterial populations during periods of starvation. mBio.

[57] Ratib N.R. (2021). Evolution in long-term stationary-phase batch culture: emergence of divergent Escherichia coli lineages over 1,200 days. mBio.

[58] Ferguson A.D., Deisenhofer J. (2004). Metal import through microbial membranes. Cell.

[59] Dixon S.J. (2012). Ferroptosis: an iron-dependent form of nonapoptotic cell death. Cell.

[60] Krewulak K.D., Vogel H.J. (2011). TonB or not TonB: is that the question?. Biochem Cell Biol.

[61] Takahashi S., Abe K., Kera Y. (2015). Bacterial d-amino acid oxidases: recent findings and future perspectives. Bioengineered.

[62] Yagi T. (1991). Bacterial NADH-quinone oxidoreductases. J Bioenerg Biomembr.

[63] Baron S.S., Terranova G., Rowe J.J. (1989). Molecular mechanism of the antimicrobial action of pyocyanin. Curr Microbiol.

[64] Rezaie J., Feghhi M., Etemadi T. (2022). A review on exosomes application in clinical trials: perspective, questions, and challenges. Cell Commun Signal.

[65] Chen K. (2021). The role of exosomes in pancreatic cancer from bench to clinical application: an updated review. Front Oncol.

[66] Sun Y., Liu J. (2014). Potential of cancer cell-derived exosomes in clinical application: a review of recent research advances. Clin Ther.

[67] Beyenal H., Chen S.N., Lewandowski Z. (2003). The double substrate growth kinetics of Pseudomonas aeruginosa. Enzym Microb Technol.

[68] Christian R.R., Manchester J.T., Mellor M.T. (1983). Bacteriological quality of fabrics washed at lower-than-standard temperatures in a hospital laundry facility. Appl Environ Microbiol.

[69] Salo S. (2000). Validation of the microbiological methods hygicult dipslide, contact plate, and swabbing in surface hygiene control: a Nordic collaborative study. J AOAC Int.

[70] Hudzicki J. (2009).

[71] Jorgensen J.H., Ferraro M.J., Antimicrobial Susceptibility Testing (2009). A review of general principles and contemporary practices. Clin Infect Dis.

[72] Auty M.A. (2001). Direct in situ viability assessment of bacteria in probiotic dairy products using viability staining in conjunction with confocal scanning laser microscopy. Appl Environ Microbiol.

[73] Honig M.G., Hume R.I. (1986). Fluorescent carbocyanine dyes allow living neurons of identified origin to Be studied in long-term cultures. JCB (J Cell Biol).

[74] Malhotra J.D. (1998). Cis-activation of L1-mediated ankyrin recruitment by TAG-1 homophilic cell adhesion. J Biol Chem.

[75] Kuriyama S. (1998). Analysis of intrahepatic invasion of hepatocellular carcinoma using fluorescent dye-labeled cells in mice. Anticancer Res.

[76] Brennan K. (2020). A comparison of methods for the isolation and separation of extracellular vesicles from protein and lipid particles in human serum. Sci Rep.

[77] Mohamed A. (2021). Hydrogen peroxide-producing electrochemical bandage controlled by a wearable potentiostat for treatment of wound infections. Biotechnol Bioeng.

[78] Karg T.J., Golic K.G. (2018). Photoconversion of DAPI and Hoechst dyes to green and red-emitting forms after exposure to UV excitation. Chromosoma.

[79] Hunter R.C., Beveridge T.J. (2005). High-resolution visualization of Pseudomonas aeruginosa PAO1 biofilms by freeze-substitution transmission electron microscopy. J Bacteriol.

