# Supplementary material for: Dual roles of the conditional extracellular vesicles derived from Pseudomonas aeruginosa biofilms: Promoting and inhibiting bacterial biofilm growth
Source: Biofilm. 2024 Feb 6;7:100183. doi: 10.1016/j.bioflm.2024.100183 (PMC10876606; doi:10.1016/j.bioflm.2024.100183)
Supplement: Multimedia component 1 [file mmc1.docx]

Supplementary Information for

**Dual Roles of the Conditional Extracellular Vesicles Derived from *Pseudomonas Aeruginosa* Biofilms: Promoting and Inhibiting Bacterial Biofilm Growth**

**Keywords:**

Bacterial extracellular vesicles, biofilm, *Pseudomonas aeruginosa,* biofilm control

**S1. Correlating the protein concentration of extracellular vesicles to the particle numbers**

To correlate the protein concentration of G-EVs to their particle numbers, the protein concentration (µg/ml) of purified extracellular vesicleswas quantified using a Pierce™ BCA Protein Assay Kit and they were stained with Vybrant™ DiI Cell-Labeling Solution (see the Materials and Methods section). The number of fluorescent extracellular vesicle particles in each image (**Fig. S1**) was correlated to the protein concentration. The protein concentration is plotted against the extracellular vesicle particle number in **Fig. S2**, which shows a linear relationship between them.

Y(number*10^3^) = 24450P(µg/ml) –458385 (R² = 0.979)


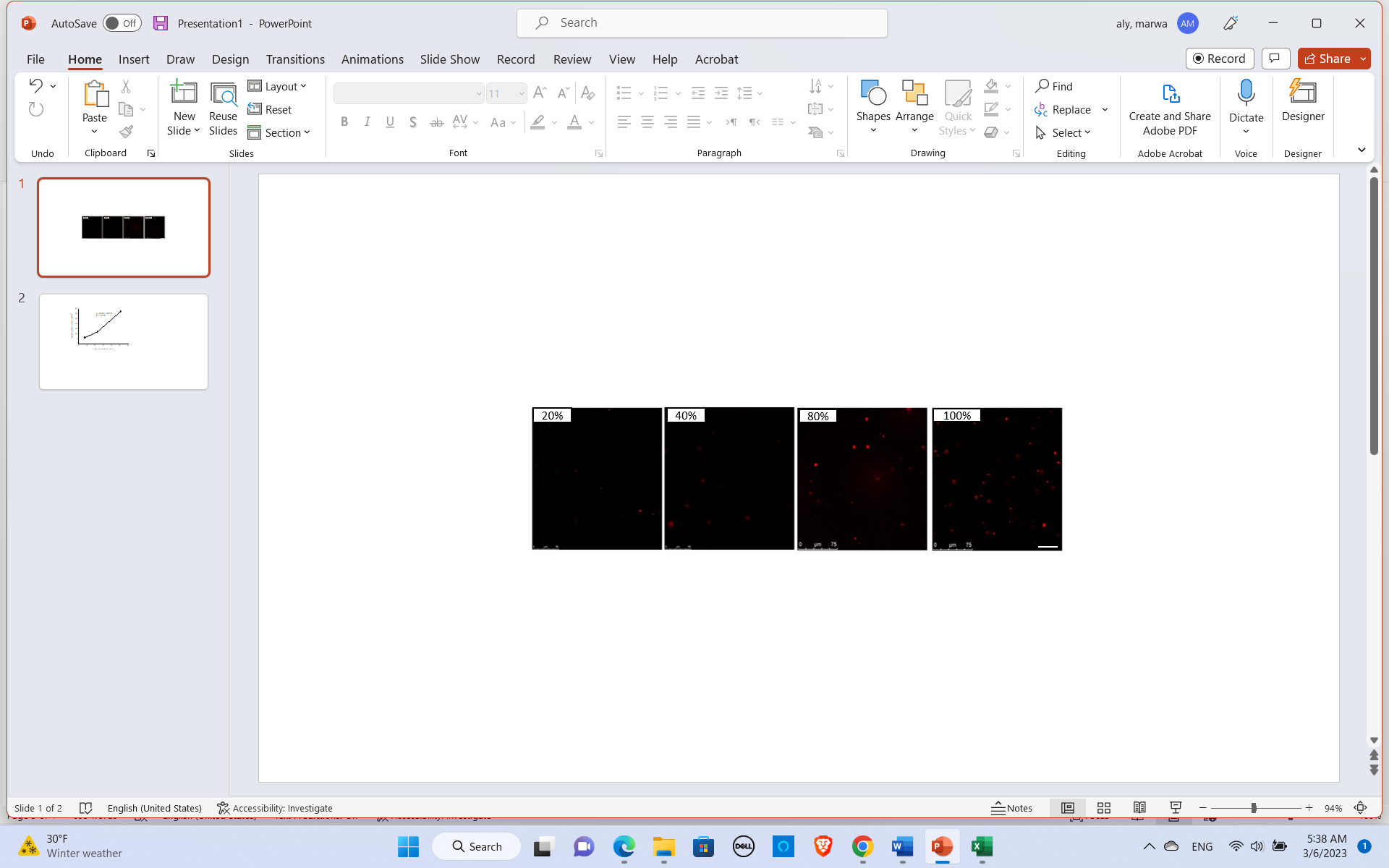
where Y is the extracellular vesicle particle number and P is the extracellular vesicle protein concentration (µg/ml). Based on this relationship, the average protein content of each extracellular vesicle particle is 0.00188 ± 8x10^-4^ µg/ml.

**Fig. S1. Quantitative fluorescence-based assay of extracellular vesicles.** Extracellular vesicles stained with Vybrant™ DiI Cell-Labeling Solution (red spots) were imaged under a confocal fluorescence microscope with an excitation/emission of 549/565 nm. The pictures from left to right show extracellular vesicle protein concentrations from low to high (20%= 26.4 ± 3 µg /ml, 40%= 40.4 ± 1 µg/ml, 80%= 78.7 ± 1 µg/ml, and 100%= 96.4 ± 4µg/ml). The scale bar indicates 75 µm.

**S2. Shape and size of extracellular vesicles**

Ten microliters of each extracellular vesicle sample were diluted with PBS at a v/v ratio of 1:1000. Four microliters of the diluted sample were then loaded onto formvar/carbon-coated copper EM grids with a thickness of 200 nm. After drying, the grids were stained with 2% uranyl acetate (4 µl) and the excess stain was immediately removed. The grids were then allowed to dry under light for 15 minutes. Finally, the grids were examined using a transmission electron microscope(FEI Tecnai G2 20 Twin equipped with a 200KV LaB6 electron source). TEM images of the purified G‑EVs and D-EVs are shown in **Fig. S3**. The extracellular vesicles extracted during the
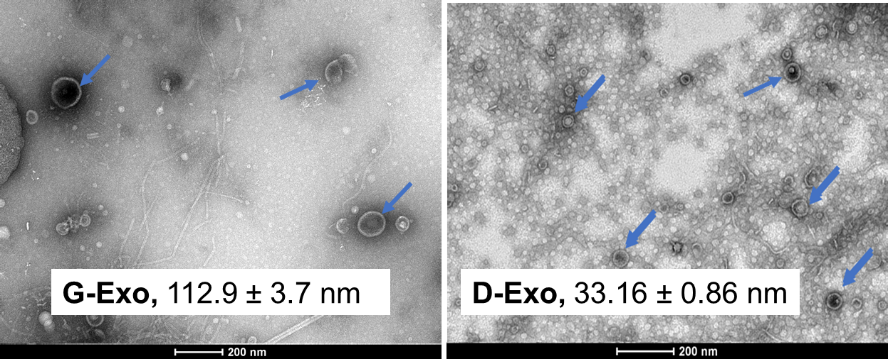
exponential growth phase werelarger (112.9 ± 3.7 nm) than theextracellular vesicles extracted during the death/survival phase (33.2 ± 0.9 nm).

**Fig. S3.** TEM images showing the sizes and shapes of the G-EVs and D-EVs from PAO1 biofilms. Samples are negatively stained with 2% uranyl acetate. Magnification power = 25000X.

**Fig. S2.** Plot of the linear relationship of extracellular vesicle protein concentration (µg/ml) to extracellular vesicle particle number (x10^3^). Data points indicate the mean, and error bars indicate standard deviations (n = 8).


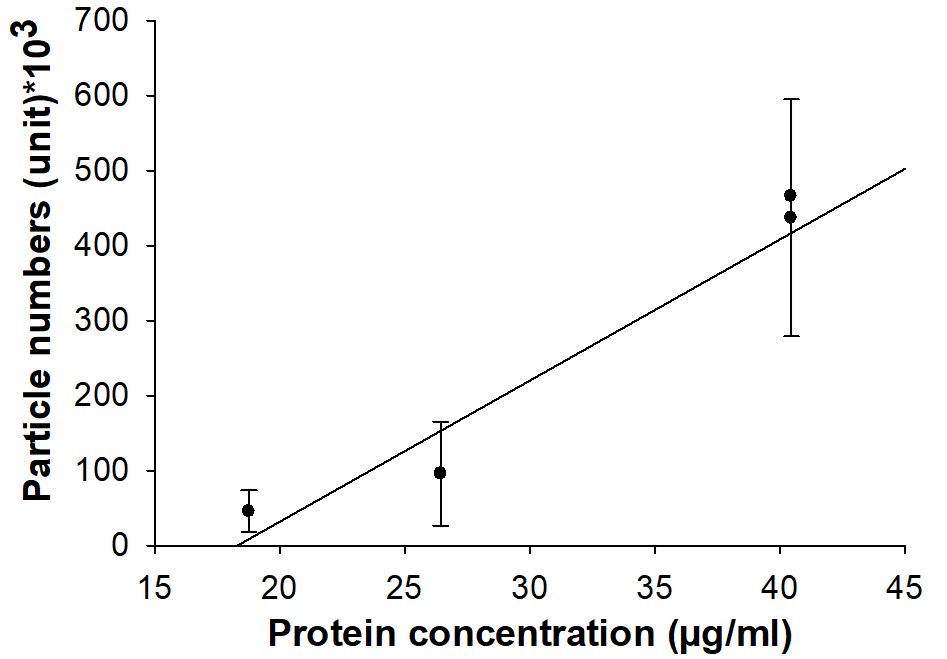


Y= 24450X–458385

R² = 0.979


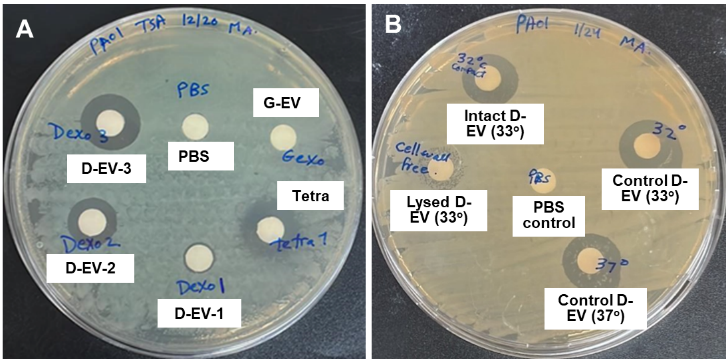
**S3. Functional effects and importance of intact extracellular vesicle structure of bacterial extracellular vesicles**

To investigate the functional effects of D-EVson PAO1 biofilm growth, we conducted a disc diffusion assay (see the Materials and Methods section) using both D-EVs and G-EVs to observe their functional effects on the growth behavior of *P. aeruginosa*PAO1 biofilm (**Fig. S4A**). PBS buffer and the antibiotic tetracycline were used as negative and positive controls, respectively, to assess the efficacy of D-EVs/G-EVs in affecting the growth of PAO1 biofilms. We used a final concentration of tetracycline of 1 µg/µl in the test to make it comparable to other treatments. G-EVs did not show an inhibition/clear zone, and D-EVs exhibited an effective and dose-dependent inhibition of PAO1 biofilm growth across a range of extracellular vesicle protein concentrations (0.11, 0.22, and 0.33 µg/µl).

**Fig. S4. A:** Kirby-Bauer disk diffusion susceptibility test of the effects of G-EVs (36.9 µg/ml) and D-EVs (1, 2, and 3 indicate 0.11, 0.22, and 0.33 µg/µl, respectively) on PAO1 colony biofilm growth. Tetracycline and PBS buffer were used as a positive control and a negative control, respectively. Note: to generate a visible clear control zone, 1 µg/µl of tetracycline was used, which is much higher than the medically tolerated doses (0.033-0.22 µg/µl) used for adult admissions [1]. **B:** Diffusion susceptibility test of the effect of the intact D-EV (33^o^) (0.33 µg/µl) and lysed D‑EV (33^o^) (0.33 µg/µl) on PAO1 colony biofilm growth. The lysed D-EV (33^o^) showed much less inhibition of biofilm growth than the intact D‑EV (33^o^). Two control D‑EV samples were also tested. D-EV(33^o^) is derived from PAO1 biofilm growing at 33^o^C, and D‑EV(37^o^) is derived from PAO1 biofilm growing at 37^o^C. D-EV(33^o^) and D‑EV(37^o^) were extracted from biofilms after 96hours of growth at 33 ^o^C and 37 °C, respectively,

Although we believe that D-EV-mediated cellular communications are key to inhibiting biofilm growth, it is possible that the observed effects are caused by toxins associated with D‑EVs, rather than by D-EV-based cellular communications. To rule out toxin-based effects and confirm that the intact extracellular vesicle structure is critical for inhibiting bacterial biofilm growth, we performed a diffusion susceptibility test to assess the inhibition effects of lysed D‑EVs. In this test, the cell walls of the extracellular vesicles were lysed by subjecting them to three 10-second water bath sonication cycles, with a 2-minute interval in ice. The results (see **Fig. S4B**) showed a clear zone of biofilm inhibition around the filter disc containing intact D-EV, whereas the zone around the disc containing lysed D-EV was much fainter. These results suggest that the intact extracellular vesicle structure, rather than toxin components, plays a role in regulating biofilm growth.

Two control D-EV samples were also tested on this plate to compare the inhibition effects of D‑EV(33^o^) and D-EV(37^o^) on PAO1 biofilm growth at 37^o^C on TSA plate (**Fig. S4 B**). The results suggest that the D-EVs derived at these two temperatures create similar inhibition zones on PAO1 biofilm plate at 37^o^C. However, close examination of the zones showed that the inhibition of D-EV(33^o^) created a clear zone, while the zone created by D-EV(37^o^) showed a few survival pathogen colonies. The reason could be that when the D-EV(37^o^) was derived from PAO1 biofilm after 96hours of growth at 37^o^C, the biofilm was still in its stationary phase (**Fig. 1**). This would make it more likely for the D-EV(37^o^) extracted under this condition to have less inhibition efficacythan the D-EV(33^o^C).

**S4. Proteomic profiles of G-EVs and D-EVs.**

Analysis of the proteomic profiles of the G-EVs and D-EVsidentified a total of 1099 and 987 proteins for G-EVs and D-EVs, respectively. Among these proteins, 79 surface and cytoplasmic proteins are shared by D-EVs and G-EVs (**Fig. S5**). The analysis also identified a total of 92 highlyabundant cytoplasmic proteins that are exclusively present in G-EVs (**Table S1**) and a total 77 highly abundant cytoplasmic proteins that are exclusively present in D-EVs (**Table S2**).

**S5. Mechanistic investigations of inhibition effects of D-EV on biofilm growth**

 To test the hypothesis that the suppression of PAO1 biofilm growth is caused by D-EV-induced excessive iron uptake by D-EV recipient cells leading to the activation of bacterial cell death, we examined whether D-EV-induced excessive iron uptake could improve the poor efficacy of the D‑EVs against 96-h biofilms (**Fig. 3D**). Briefly, we first treated 96-h PAO1 biofilms with 2 doses of D-EV at a protein concentration of 0.33 µg/µL with a 12-hour interval, which only induced an
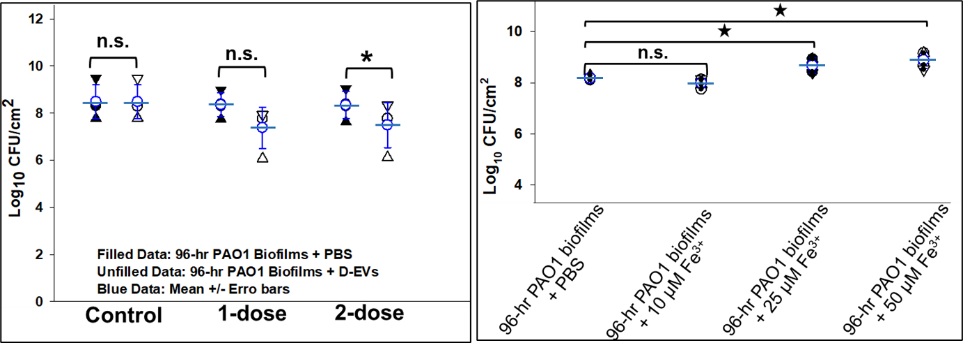
inhibition of less than 1-log_10_ (**Fig. S6A**). We then repeated the same inhibition experi­ments applying 10 µM, 25µ M, or 50 µM of Fe^3+^ directly to the 96-h PAO1 biofilms to see how iron alone affects the growth of 96-h PAO1 biofilms. Compared to the PBS control, the presence of ferric ions alone marginally promoted biofilm growth with a dose-dependent effect (**Fig. S6B**).

**Fig. S6**. **A:** Inhibition effects of D-EVs on 96-h PAO1 biofilms. This graph is the same asFig. 3D, and it is shown here for comparison to Fig. S6B. Two doses of D-EVs at a protein concentration of 0.33 µg/µL were applied to the biofilms with a 12-hour interval, which induced an inhibition of less than 1‑log_10_. **B:** Effects of 10 µM, 25 µM and 50 µM of ferric ions on 96-h biofilm. For all data points, biofilms were collected and analyzed 24 hours after each treatment. Ferric ions alone showed a very minimal effect on the biofilm growth. The mean value and error bar of each group of data are given in blue. P-value <0.05 (*). n.s.: not significant.

**S6. Cell viability of D-EVs derived from PAO1 biofilms**

We investigated the toxicity of PAO1 D-EVs (0.33 µg/µl) to mammalian cell viability. Human mesenchymal stem cells (hMSC) obtained from ATCC (Manassas, VA) were cultured in alpha-MEM medium with 5% FBS, 1% L-glutamine, and 1% Pen-Strep in an incubator with 5% CO_2_ at 37°C. Cells were then used to seed 6-well plates with an inoculation rate of 10^4 and cultured to 60-70% confluency before being exposed to D-EV (0.33 µg/µl) or PBS (as a control) in separate cultures of hMSC. After 24 hours and 48 hours of exposure, the growth and cellular morphologies of the hMSC were examined. The results, depicted in **Fig. S7A**and **Fig. S7B**, showed no significant detrimental effects on either cell growth or the cellular morphology of hMSC after two days of exposure to D-EVs. These results demonstrate that the presence of D-EVs does not affect cellular functions and thus provide a foundation for exploring the potential use of D-EVs in antibiotic applications.


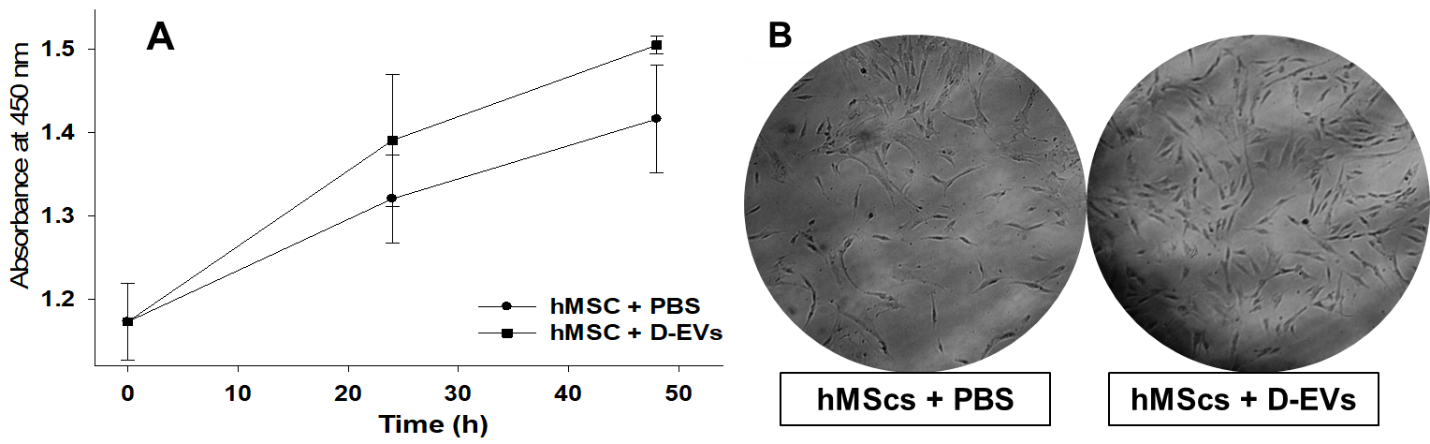


**Fig. S7. Cytotoxicity test of D-EVs to human mesenchymal stem cell culture. A.** Changes in hMSC growth in the absence/presence of D-EVs (0.33 µg/µl) were monitored for two days. At each time point, cells were stained with Cell Proliferation Reagent WST-1 and incubated for four hours. The absorbance was measured at 450 nm to monitor the replication of their genomic DNA. **B.**Light microscopy images showing changes in cellular morphology of hMSCs 48 hours after treatment with D-EVs (0.33 µg/µl).
