# Supplementary material for: Dual roles of the conditional extracellular vesicles derived from Pseudomonas aeruginosa biofilms: Promoting and inhibiting bacterial biofilm growth
Source: Biofilm. 2024 Feb 6;7:100183. doi: 10.1016/j.bioflm.2024.100183 (PMC10876606; doi:10.1016/j.bioflm.2024.100183)
Supplement: Multimedia component 2 [file mmc2.docx]

**Table S1. Summary of proteomic profiles of cytoplasmic proteins of G-EVs**

| Accession | Description | | | Sum PEP Score | Coverage [%] | | # of PSMs | | # Unique Peptides | # AAs | MW [kDa] | Abundances |
| --- | --- | --- | --- | --- | --- | --- | --- | --- | --- | --- | --- | --- |
| **Growth promotors** | |  |  |  |  |  | |  | |  |  |  |
| A0A653B731 | Cell division proteins | | | 6.093 | 4 | | 1 | | 1 | 398 | 41.7 | 100 |
| V6A9F4 | Phasin_2 domain-containing protein | | | 1.275 | 12 | | 2 | | 1 | 153 | 16.9 | 34.64 |
| A0A1C7B9B8 | Elongation factors | | | 48.011 | 23 | | 22 | | 11 | 706 | 77.7 | 58.34 |
| A0A7W3UUK1 | Chaperonins | | | 11.74 | 10 | | 7 | | 1 | 527 | 55.7 | 32.5 |
| A0A3D9EJG5 | Chaperone(s) | | | 1.443 | 4 | | 1 | | 1 | 433 | 47.9 | 35.28 |
| **DNA synthesis** | |  |  |  |  |  |  |  |  |  |  |  |
| A0A072ZFF8 | N5-carboxyaminoimidazole ribonucleotide mutase | | | 37.121 | 67 | | 22 | | 5 | 163 | 16.9 | 60.32 |
| A0A086BUQ2 | Vitamin B12-dependent ribonucleotide reductase | | | 14.186 | 7 | | 4 | | 3 | 734 | 82.7 | 64.4 |
| S6ABT0 | Ribonucleotide reductase | | | 3.464 | 3 | | 3 | | 1 | 324 | 35.6 | 33.98 |
| A0A3M5EFC0 | Polyribonucleotide nucleotidyltransferase | | | 116.971 | 34 | | 60 | | 16 | 748 | 80.3 | 49.36 |
| A0A367M4C8 | HU family DNA-binding protein | | | 27.865 | 49 | | 19 | | 5 | 93 | 9.8 | 68.58 |
| A0A069PX19 | DNA polymerases | | | 18.819 | 11 | | 7 | | 6 | 913 | 99.7 | 61.2 |
| A0A086BZZ8 | DNA topoisomerase | | | 13.875 | 10 | | 6 | | 5 | 868 | 97.2 | 56.28 |
| Q9I3X2 | DNA helicase | | | 7.812 | 6 | | 3 | | 3 | 711 | 79.8 | 41.14 |
| A0A485FIC3 | DNA gyrase | | | 41.307 | 11 | | 18 | | 6 | 925 | 101.3 | 43 |
| A0A2S5IJE8 | Holliday junction ATP-dependent DNA helicase | | | 1.637 | 6 | | 1 | | 1 | 205 | 22.3 | 69.12 |
| **Protein processing** | |  |  |  |  |  | | | |  |  |  |
| A0A086C2F0 | Peptide-binding protein | | | 9.811 | 18 | | 3 | | 3 | 222 | 24.1 | 100 |
| A0A397MC94 | SsrA-binding protein | | | 2.281 | 6 | | 1 | | 1 | 197 | 22.3 | 100 |
| A4XZJ8 | Putative serine protein kinase | | | 14.827 | 11 | | 9 | | 1 | 640 | 73.7 | 100 |
| A0A485EER1 | NAD-capped RNA hydrolase | | | 3.84 | 2 | | 1 | | 1 | 841 | 92.3 | 100 |
| A0A367M033 | 2-oxo-4-hydroxy-4-carboxy-5-ureidoimidazoline decarboxylase | | | 3.647 | 9 | | 1 | | 1 | 178 | 19.6 | 100 |
| A0A080VRC4 | S-adenosylmethionine:tRNA ribosyltransferase-isomerase | | | 3.558 | 4 | | 1 | | 1 | 347 | 38.1 | 100 |
| A0A5E9K219 | Ribosomal RNA large subunit methyltransferase H | | | 2.577 | 12 | | 2 | | 1 | 155 | 17.8 | 100 |
| A0A072ZEM5 | Aminopeptidase P family protein | | | 2.244 | 3 | | 1 | | 1 | 405 | 44.1 | 100 |
| A0A0H2ZCX7 | Putative MoxR protein O | | | 2.446 | 4 | | 1 | | 1 | 305 | 32.8 | 100 |
| A0A1I1XAM6 | Adenylyltransferase and sulfurtransferase | | | 1.941 | 4 | | 1 | | 1 | 270 | 28.4 | 100 |
| W1MVH1 | Glutathione S-transferase | | | 1.38 | 5 | | 1 | | 1 | 256 | 28.8 | 100 |
| A0A0A8RCC8 | Periplasmic tail-specific protease | | | 2.185 | 1 | | 1 | | 1 | 840 | 93.5 | 100 |
| A0A072ZJB8 | Carboxy-terminal processing protease precurs | | | 2.716 | 8 | | 2 | | 2 | 436 | 46 | 31.7 |
| B7V669 | 50S ribosomal proteins | | | 56.677 | 49 | | 26 | | 7 | 129 | 14.5 | 42.56 |
| A0A1C7C943 | 30S ribosomal proteins | | | 35.017 | 28 | | 26 | | 4 | 246 | 27.3 | 33.62 |
| A0A231K3D3 | Chain-length determining protein | | | 16.217 | 17 | | 5 | | 4 | 442 | 49.2 | 8.08 |
| W1MWV0 | Glycine--tRNA ligase alpha subunit | | | 13.843 | 24 | | 7 | | 5 | 318 | 36.5 | 42.18 |
| A0A0C7D284 | Tyrosine--tRNA ligase | | | 6.676 | 10 | | 2 | | 2 | 399 | 44.1 | 80.42 |
| A0A0A8RE97 | Leucine--tRNA ligase | | | 6.149 | 3 | | 4 | | 2 | 873 | 97.6 | 30.8 |
| Q9HXU0 | Lysine--tRNA ligase | | | 11.043 | 12 | | 4 | | 4 | 501 | 57.3 | 72.02 |
| A0A0F6RS41 | Phenylalanine--tRNA ligase | | | 1.284 | 2 | | 1 | | 1 | 792 | 86.7 | 61.18 |
| A0A367MBX4 | Glutamine--tRNA ligase | | | 7.116 | 8 | | 4 | | 3 | 561 | 63.3 | 83.14 |
| L8MK56 | Glutamyl-tRNA(Gln) amidotransferase subunit | | | 4.254 | 6 | | 1 | | 1 | 483 | 51.4 | 58.26 |
| A0A2R4BHU9 | Aspartyl/glutamyl-tRNA(Asn/Gln) amidotransferase subunit | | | 3.26 | 3 | | 3 | | 1 | 492 | 54.6 | 50.4 |
| A0A485HEP7 | D-aminoacyl-tRNA deacylase | | | 3.839 | 4 | | 1 | | 1 | 468 | 51.9 | 86.76 |
| A0A1H0JDB1 | Succinate--CoA ligase | | | 25.501 | 17 | | 9 | | 6 | 388 | 41.5 | 68.46 |
| A0A0A8RKE2 | Isoleucine--tRNA ligase | | | 42.217 | 14 | | 23 | | 9 | 943 | 105.4 | 52.02 |
| A6V3C2 | Ribonuclease | | | 11.6 | 5 | | 4 | | 3 | 1073 | 119.3 | 49.66 |
| A0A086C201 | RNA polymerase | | | 8.959 | 6 | | 5 | | 1 | 620 | 70.1 | 53.84 |
| A0A087L9F7 | Alkaline phosphatase family protein | | | 7.665 | 12 | | 3 | | 2 | 269 | 30 | 59.9 |
| A0A2R3ISX8 | Bifunctional proteins | | | 6.982 | 13 | | 3 | | 3 | 454 | 48.8 | 83.18 |
| A0A1G8LM51 | RNA-binding protein | | | 23.925 | 66 | | 18 | | 6 | 86 | 9.5 | 43.04 |
| A0A0A8RIM8 | Chitin-binding protein | | | 17.973 | 28 | | 12 | | 6 | 389 | 41.8 | 46.38 |
| A0A0D6INW2 | Ribosome-binding factor | | | 10.607 | 32 | | 4 | | 3 | 130 | 14.7 | 56.04 |
| A0A127MQF6 | Ribosome modulation factor | | | 5.574 | 21 | | 3 | | 1 | 72 | 8.4 | 46.14 |
| A0A2R3INC8 | Ribosome-recycling factor | | | 15.315 | 22 | | 10 | | 3 | 185 | 20.5 | 86.18 |
| A0A1C7BK43 | Periplasmic serine endoprotease | | | 1.523 | 3 | | 1 | | 1 | 474 | 50.3 | 65.86 |
| A0A3M5DFV1 | ATP-dependent Clp protease ATP-binding subunit | | | 13.897 | 8 | | 7 | | 2 | 452 | 50.1 | 67.32 |
| A0A069PZV2 | Enoyl-[acyl-carrier-protein] reductase [NADH] | | | 1.652 | 6 | | 1 | | 1 | 265 | 28 | 73.34 |
| K7Y4J0 | Alkaline metalloprotease | | | 9.962 | 7 | | 5 | | 2 | 481 | 50.6 | 59.52 |
| A0A072ZPJ9 | Metalloprotease | | | 7.361 | 6 | | 2 | | 2 | 449 | 47.8 | 70.46 |
| A6VCK8 | ATP-dependent zinc metalloprotease | | | 6.179 | 2 | | 2 | | 1 | 642 | 70.3 | 43.9 |
| A0A0A8RQS8 | Zn_protease domain-containing protein | | | 10.585 | 20 | | 7 | | 3 | 221 | 24.3 | 67.76 |
| A0A1I1UAD0 | Ferritin-like metal-binding proteins | | | 5.342 | 8 | | 3 | | 1 | 169 | 18.8 | 63.86 |
| **Flagella and Pilli** | |  |  |  |  | | | | |  |  |  |
| A0A071KYA9 | Flagellar biosynthesis protein | | | 6.771 | 17 | | 2 | | 2 | 156 | 17.2 | 88.96 |
| A0A0A8RCK3 | Protein pilG | | | 4.435 | 13 | | 1 | | 1 | 135 | 14.7 | 80.94 |
| A0A379IZN6 | Type IV-A pilus assembly ATPase | | | 1.889 | 4 | | 2 | | 1 | 581 | 63.8 | 72.98 |
| Q9HVM8 | Type IV pilus biogenesis factor PilY1 | | | 56.48 | 22 | | 23 | | 12 | 1161 | 126.5 | 33.6 |
| A0A0A8RBR3 | Protein pilJ | | | 20.55 | 9 | | 10 | | 4 | 682 | 72.5 | 30.84 |
| Synthases | |  |  |  |  | | | | |  |  |  |
| A0A3M5EVT4 | 3-dehydroquinate synthase OS=Pseudomonas aeruginosa OX=287 GN=aroB PE=3 SV=1 | | | 5.761 | 9 | | 2 | | 2 | 421 | 46.1 | 100 |
| A0A069Q8Q1 | 1,4-Dihydroxy-2-naphthoyl-CoA synthase OS=Pseudomonas aeruginosa OX=287 GN=menB_1 PE=3 SV=1 | | | 5.632 | 7 | | 1 | | 1 | 265 | 28.9 | 100 |
| A0A291KBQ0 | S-adenosylmethionine synthase OS=Pseudomonas mendocina OX=300 GN=metK PE=3 SV=1 | | | 5.509 | 7 | | 2 | | 1 | 396 | 42.7 | 100 |
| A0A0A8RA44 | Acetolactate synthase OS=Pseudomonas aeruginosa OX=287 GN=PAMH19_0813 PE=3 SV=1 | | | 4.099 | 3 | | 2 | | 1 | 592 | 64.7 | 100 |
| A0A0A8RC77 | Anthranilate synthase component 1 OS=Pseudomonas aeruginosa OX=287 GN=trpE PE=3 SV=1 | | | 2.933 | 4 | | 1 | | 1 | 496 | 55.1 | 100 |
| A0A3D9EI68 | ATP synthase | | | 130.604 | 42 | | 77 | | 1 | 458 | 49.7 | 37.78 |
| A0A0A8RS72 | Hydrogen cyanide synthase | | | 33.235 | 29 | | 10 | | 8 | 464 | 50.4 | 83.46 |
| A0A077JN20 | GMP synthase | | | 9.213 | 8 | | 5 | | 3 | 527 | 58.1 | 45.4 |
| A0A0A8RJH1 | Glucans biosynthesis glucosyltransferase | | | 8.951 | 7 | | 4 | | 4 | 861 | 97 | 91.06 |
| A0A127MNP8 | Argininosuccinate synthase | | | 8.785 | 5 | | 4 | | 2 | 405 | 45.4 | 73.16 |
| A0A0F6UIJ7 | Carbamoyl-phosphate synthase large chain | | | 8.595 | 3 | | 3 | | 2 | 1073 | 117.3 | 66.24 |
| A0A081HI22 | Tryptophan synthase | | | 8.141 | 17 | | 2 | | 2 | 268 | 28.5 | 84.78 |
| A0A0A8RJ65 | PQB biosynthetic 3-oxoacyl-[acyl-carrier-protein] synthase | | | 7.385 | 7 | | 5 | | 2 | 348 | 37.6 | 69.48 |
| A0A5K1SNI7 | Chorismate synthase | | | 5.676 | 8 | | 2 | | 2 | 363 | 38.9 | 33.44 |
| A0A127MLM1 | Thiazole synthase | | | 5.568 | 11 | | 3 | | 2 | 268 | 28.5 | 42.72 |
| A0A6H3G8H9 | Lipopolysaccharide biosynthesis protein | | | 5.095 | 7 | | 3 | | 2 | 662 | 74.5 | 64.84 |
| A0A0A8RHR0 | Acetolactate synthase | | | 4.525 | 3 | | 2 | | 1 | 574 | 63 | 90.44 |
| A0A0A8RG31 | Dihydrofolate synthase/folylpolyglutamate synthase | | | 4.132 | 4 | | 2 | | 1 | 429 | 46.5 | 61.64 |
| A0A3M5DAV0 | Biotin synthase | | | 1.038 | 3 | | 1 | | 1 | 390 | 43.3 | 85.78 |
| A0A3M5ENA3 | Citrate synthase | | | 96.305 | 62 | | 53 | | 14 | 429 | 47.8 | 51.02 |
| A0A0A8RPU8 | Phosphoenolpyruvate synthase | | | 55.493 | 22 | | 34 | | 13 | 791 | 85.8 | 44.82 |
| A0A0C6EL94 | Malate synthase | | | 45.881 | 29 | | 17 | | 9 | 725 | 78.6 | 55.46 |
| A0A1C7BDZ1 | Pyridoxine 5'-phosphate synthase | | | 29.474 | 27 | | 18 | | 5 | 248 | 27.2 | 44.54 |
| A0A086BTL8 | Adenylosuccinate synthetase | | | 29.298 | 27 | | 16 | | 7 | 430 | 46.8 | 45.76 |
| A0A069QI07 | Glutathione synthetase | | | 21.722 | 25 | | 8 | | 4 | 317 | 35.7 | 34.52 |
| **Oxidoreductase enzymes** | |  |  |  |  |  | |  | |  |  |  |
| A0A5F1BV16 | Re/Si-specific NAD(P)(+) transhydrogenase | | | 8.376 | 10 | | 3 | | 2 | 373 | 38.8 | 100 |
| A0A0H2Z7C1 | N-succinylglutamate 5-semialdehyde dehydrogenase | | | 5.799 | 4 | | 1 | | 1 | 488 | 51.5 | 100 |
| A0A485EKG2 | Gluconate dehydrogenase | | | 5.047 | 3 | | 3 | | 2 | 1275 | 138.2 | 100 |
| A0A7U9F023 | GDP-mannose 6-dehydrogenase | | | 2.742 | 3 | | 1 | | 1 | 442 | 48.2 | 100 |
|  |  | | |  |  | |  | |  |  |  |  |

**Note:**

# PSMs (the total number of identified peptide spectra matched for the protein)

FDR Confidence Combined was high for all proteins.

Exp. q-value: Combined was between 0-0.004
