# Supplementary material for: Dual roles of the conditional extracellular vesicles derived from Pseudomonas aeruginosa biofilms: Promoting and inhibiting bacterial biofilm growth
Source: Biofilm. 2024 Feb 6;7:100183. doi: 10.1016/j.bioflm.2024.100183 (PMC10876606; doi:10.1016/j.bioflm.2024.100183)
Supplement: Multimedia component 3 [file mmc3.docx]

**Table S2. Summary of proteomic profiles of cytoplasmic proteins of D-EVs**

| Accession | Description | | | Sum PEP Score | | Coverage [%] | | | # PSMs | | # Unique Peptides | | | # AAs | | MW [kDa] | Abundance (%) | |
| --- | --- | --- | --- | --- | --- | --- | --- | --- | --- | --- | --- | --- | --- | --- | --- | --- | --- | --- |
| **Fatty acid oxidation and hydrogen peroxide production** | | | | | |  | | |  |  |  |  |  |  |  |  |  |  |
| A0A1C7BKA1 | DAO domain-containing protein | | | 6.348 | | 6 | | | 2 | | 2 | | | 468 | | 52.2 | 44.44 | |
| A0A2R3QM54 | D-amino acid dehydrogenase | | | 3.474 | | 10 | | | 3 | | 1 | | | 432 | | 47 | 100 | |
| A0A127MPZ7 | Soluble pyridine nucleotide transhydrogenase | | | 112.322 | | 57 | | | 94 | | 1 | | | 464 | | 51.3 | 72.72 | |
| A0A1H0H0K0 | 2-hydroxycyclohexanecarboxyl-CoA dehydrogenase | | | 1.326 | | 4 | | | 2 | | 1 | | | 255 | | 26.1 | 67.18 | |
| A0A080VW75 | CatB-related O-acetyltransferase | | | 1.726 | | 7 | | | 1 | | 1 | | | 229 | | 25.6 | 65.42 | |
| A0A069QJT4 | Glycerophosphodiester phosphodiesterase | | | 23.456 | | 26 | | | 10 | | 4 | | | 240 | | 26.9 | 31.98 | |
| A0A080VGG0 | Acetyl-coenzyme A synthetase | | | 9.765 | | 5 | | | 3 | | 2 | | | 645 | | 71.6 | 35.4 | |
| A0A1G8E7R7 | Glutamate synthase (NADPH/NADH) large chain | | | 4.786 | | 1 | | | 1 | | 1 | | | 1482 | | 161.9 | 54.66 | |
| A0A0A8RMZ0 | Dihydrolipoamide acetyltransferase component of pyruvate dehydrogenase complex | | | 23.135 | | 6 | | | 10 | | 2 | | | 428 | | 45.5 | 42.34 | |
| **Protein oxidation and activate the Fenton reaction** | | | |  | | | | | | | |  |  |  |  |  |  |  |
| A0A7U0JSC5 | AAA family ATPase | | | 6.038 | | 5 | | | 3 | | 2 | | | 555 | | 57.7 | 80 | |
| A0A3M5EPG2 | Dihydroxy-acid dehydratase | | | 2.593 | | 2 | | | 2 | | 1 | | | 680 | | 72.7 | 39.26 | |
| A0A3M5DZ62 | OMP_b-brl domain-containing protein | | | 60.174 | | 42 | | | 27 | | 10 | | | 236 | | 25.6 | 53.68 | |
| A0A3M5D221 | STN domain-containing protein | | | 5.527 | | 2 | | | 2 | | 1 | | | 977 | | 105.6 | 71.16 | |
| A0A1H0IZF5 | LPS-assembly protein LptD | | | 1.195 | | 1 | | | 1 | | 1 | | | 912 | | 102.8 | 57.06 | |
| A0A3D9E7A8 | Urease accessory protein UreF | | | 1.136 | | 10 | | | 1 | | 1 | | | 223 | | 24.4 | 77.22 | |
| A0A5F1BVL6 | YkgJ family cysteine cluster protein | | | 1.281 | | 15 | | | 1 | | 1 | | | 223 | | 24.8 | 81.88 | |
| A0A0A8RCJ6 | UvrABC system protein A | | | 13.698 | | 7 | | | 5 | | 5 | | | 1003 | | 110.3 | 42.78 | |
| A0A1G9YY22 | Sterol carrier protein | | | 6.833 | | 16 | | | 1 | | 1 | | | 104 | | 11.1 | 100 | |
| A0A0A8RGJ4 | Protein PelC | | | 7.15 | | 12 | | | 2 | | 1 | | | 172 | | 18.6 | 72.7 | |
| A0A072ZPD2 | Amino-acid carrier protein AlsT | | | 10.903 | | 6 | | | 2 | | 1 | | | 449 | | 47.3 | 31.46 | |
| W1MFI8 | Lactamase_B domain-containing protein | | | 8.994 | | 7 | | | 5 | | 2 | | | 433 | | 48.6 | 41.32 | |
| A0A0A8RHK2 | MaoC-like domain-containing protein | | | 10.152 | | 19 | | | 4 | | 3 | | | 285 | | 31.1 | 39.32 | |
| A0A080VTX5 | Putative periplasmic transport protein | | | 8.078 | | 11 | | | 4 | | 2 | | | 319 | | 33.6 | 64.1 | |
| A0A086C084 | Terminase OS=Pseudomonas aeruginosa VRFPA01 | | | 3.309 | | 9 | | | 1 | | 1 | | | 119 | | 13.3 | 30.72 | |
| **Unfold DNA, RNA, and proteins** | | | |  | | | | | | | | | |  |  |  |  |  |
| A0A0A8RE90 | ATP-dependent RNA helicase RhlB | | | 3.444 | | 2 | | | 1 | | 1 | | | 579 | | 63.8 | 65 | |
| A0A072ZF84 | 3-guanidinopropionase | | | 5.869 | | 6 | | | 2 | | 1 | | | 318 | | 34.2 | 57.04 | |
| **Promote cellular adhesion** | |  |  | |  |  | |  | |  | | | |  |  |  |  |  |
| A0A367LV53 | Aldehyde dehydrogenase family protein (Fragment) | | | 1.788 | | 11 | | | 2 | | 1 | | | 99 | | 11 | 100 | |
| A0A2R3IQ67 | Neisseria PilC beta-propeller domain protein | | | 6.646 | | 3 | | | 2 | | 1 | | | 1155 | | 126.1 | 100 | |
| A0A1I1TGJ6 | Flagellar L-ring protein | | | 3.893 | | 8 | | | 1 | | 1 | | | 237 | | 24.9 | 100 | |
| A0A485GA02 | Type 4 fimbrial biogenesis protein PilW | | | 11.1 | | 11 | | | 4 | | 3 | | | 625 | | 68.7 | 30.4 | |
| A0A0F6UFV5 | Flagellar hook-associated protein 3 | | | 121.787 | | 60 | | | 63 | | 16 | | | 439 | | 46.8 | 47.68 | |
| A0A1C7BE42 | Fimbrial assembly protein pilQ | | | 33.737 | | 21 | | | 22 | | 9 | | | 714 | | 77.4 | 68.1 | |
| A0A022P6S5 | Flagellar basal-body rod protein FlgG | | | 31.805 | | 39 | | | 17 | | 7 | | | 261 | | 27.7 | 40.98 | |
| A0A072ZBU1 | Fap amyloid fiber secretin | | | 5.721 | | 8 | | | 3 | | 2 | | | 421 | | 45.7 | 40.02 | |
| A0A3M5EU50 | Flagellar P-ring protein | | | 6.05 | | 6 | | | 3 | | 2 | | | 554 | | 57.4 | 90.62 | |
| **Virulence factors** | |  |  | |  |  | |  | |  | | | |  |  |  |  |  |
| A0A7Z0KFT4 | Flagellin | | | 6.102 | | 12 | | | 5 | | 1 | | | 397 | | 40.6 | 100 | |
| A0A6M5KA93 | B-type flagellin | | | 74.411 | | 84 | | | 74 | | 7 | | | 126 | | 13.3 | 92.02 | |
| **Catalyzes** | | | |  | | |  | |  | | | |  | |  |  | |  |
| A0A0A8R9J6 | Hydrolase_4 domain-containing protein | | | 4.573 | | 7 | | | 2 | | 2 | | | 339 | | 38.3 | 100 | |
| A0A072ZRC9 | Nucleotide sugar epimerase/dehydratase WbpM | | | 2.571 | | 2 | | | 1 | | 1 | | | 665 | | 74.3 | 100 | |
| A0A5E5R2E7 | Esterase EstA | | | 116.039 | | 43 | | | 74 | | 2 | | | 646 | | 69.6 | 74.84 | |
| A0A7U9F3D4 | Transaldolase | | | 5.763 | | 7 | | | 3 | | 1 | | | 309 | | 33.9 | 34.6 | |
| A0A7U4A4H5 | Soluble pyridine nucleotide transhydrogenase | | | 170.703 | | 63 | | | 138 | | 5 | | | 464 | | 51.1 | 66.72 | |
| V6AM38 | Carbamoyl-phosphate synthase small chain | | | 2.158 | | 3 | | | 1 | | 1 | | | 398 | | 43.2 | 100 | |
| Q9L6C7 | Triacylglycerol acylhydrolase | | | 137.528 | | 59 | | | 114 | | 3 | | | 311 | | 32.7 | 41.98 | |
| A0A0H2ZGA2 | RND efflux membrane fusion protein | | | 40.982 | | 31 | | | 19 | | 7 | | | 370 | | 39.1 | 51.02 | |
| A0A1C7B566 | Lysin domain-containing protein | | | 40.492 | | 21 | | | 22 | | 7 | | | 341 | | 37.6 | 75.92 | |
| A0A077JXU0 | Alpha/beta hydrolase | | | 1.343 | | 4 | | | 2 | | 1 | | | 275 | | 30.4 | 46.26 | |
| A0A643IQV2 | Protocatechuate 3,4-dioxygenase subunit beta | | | 7.402 | | 10 | | | 1 | | 1 | | | 239 | | 27.2 | 100 | |
| A0A0A8RRW3 | Uricase | | | 3.874 | | 6 | | | 2 | | 2 | | | 494 | | 55 | 57.36 | |
| A0A485EQT2 | Signal peptidase I | | | 6.5 | | 16 | | | 2 | | 2 | | | 214 | | 24 | 40.88 | |
| A0A1C7BZ49 | Lipoprotein | | | 6.93 | | 7 | | | 5 | | 1 | | | 210 | | 23.7 | 56.8 | |
| A0A086BXD4 | Peptidyl-tRNA hydrolase | | | 1.248 | | 7 | | | 1 | | 1 | | | 194 | | 20.8 | 100 | |
| **Cellular permeability** | |  |  | |  |  | |  | |  | | | |  |  |  |  |  |
| A0A086BU08 | ABC transporter permease | | | 1.279 | | 3 | | | 2 | | 1 | | | 231 | | 24.9 | 54.84 | |
| A0A3S0IYB5 | Autotransporter domain-containing protein | | | 104.446 | | 21 | | | 83 | | 1 | | | 991 | | 104.4 | 41.58 | |
| A0A0H2Z999 | Putative outer membrane receptor protein | | | 51.845 | | 24 | | | 19 | | 11 | | | 705 | | 79.2 | 33.32 | |
| Q9HVG7 | POTRA domain-containing proteinX=208964 GN=PA4624 PE=3 SV=1 | | | 101.304 | | 54 | | | 57 | | 18 | | | 568 | | 63.2 | 37.78 | |
| A0A7M3A5L5 | Porins | | | 71.452 | | 59 | | | 47 | | 1 | | | 204 | | 22.9 | 100 | |
| A0A0A8RIR9 | ABC transporter ATP-binding protein/permease | | | 2.707 | | 2 | | | 1 | | 1 | | | 682 | | 74.7 | 100 | |
| A0A6N4IRW4 | Type VI secretion system contractile sheath large subunit | | | 23.3 | | 18 | | | 11 | | 5 | | | 491 | | 55.5 | 59.14 | |
| A0A3M5E7S2 | ATP-grasp domain-containing protein | | | 23.138 | | 21 | | | 9 | | 7 | | | 519 | | 59.5 | 64.08 | |
| A0A072ZK48 | Outer membrane lipoprotein Blc | | | 1.537 | | 5 | | | 1 | | 1 | | | 189 | | 22 | 14.24 | |
| A0A080VSF7 | Pseudopaline transport outer membrane protein CntO | | | 11.172 | | 8 | | | 3 | | 3 | | | 708 | | 79 | 55.96 | |
| **Regulators** | | | |  | | |  | |  | | | |  | |  |  | |  |
| A0A6A9JUZ1 | Response regulator | | | 3.509 | | 9 | | | 1 | | 1 | | | 212 | | 23.2 | 100 | |
| A0A1H0PGG6 | Soluble pyridine nucleotide transhydrogenase | | | 77.621 | | 33 | | | 68 | | 2 | | | 464 | | 51.1 | 85.7 | |
| A0A7Y9XNH5 | Type I restriction enzyme R subunit | | | 1.097 | | 3 | | | 1 | | 1 | | | 910 | | 103.1 | 100 | |
| A0A5R1AUH1 | TetR family transcriptional regulator | | | 2.007 | | 4 | | | 1 | | 1 | | | 212 | | 24 | 39.9 | |
| A0A069QCL6 | Protocatechuate 3,4-dioxygenase alpha chain | | | 2.443 | | 7 | | | 2 | | 1 | | | 201 | | 22.8 | 53.08 | |
| A0A086BV97 | Transcriptional regulator | | | 2.769 | | 5 | | | 1 | | 1 | | | 225 | | 25.5 | 56.6 | |
| V6AMV0 | Putative methyl-accepting chemotaxis protein | | | 13.017 | | 3 | | | 4 | | 1 | | | 859 | | 91.1 | 63 | |
| A0A0A8RAB2 | Putative HTH-type transcriptional regulator YdcR | | | 14.153 | | 11 | | | 6 | | 4 | | | 500 | | 55.3 | 100 | |
| A0A0D7MRK9 | Chemotaxis transducer | | | 16.779 | | 12 | | | 4 | | 3 | | | 545 | | 58.3 | 71.76 | |
| A0A0A8RJ29 | HIT domain-containing protein | | | 4.547 | | 7 | | | 1 | | 1 | | | 209 | | 23 | 69.86 | |
| A0A3M5ED98 | HTH tetR-type domain-containing protein | | | 3.801 | | 4 | | | 1 | | 1 | | | 274 | | 30 | 100 | |
| A0A3D9E778 | Translation initiation factor IF-2 | | | 3.756 | | 2 | | | 1 | | 1 | | | 852 | | 91.9 | 39.32 | |
| V6A9P6 | PhoH-like protein | | | 2.99 | | 5 | | | 1 | | 1 | | | 363 | | 41.2 | 69.26 | |
| A0A7U3TKQ8 | HAMP domain-containing protein | | | 8.311 | | 3 | | | 1 | | 1 | | | 575 | | 62.2 | 100 | |
| A0A3M5ELG9 | Protein translocase subunit SecD | | | 30.446 | | 18 | | | 11 | | 8 | | | 622 | | 67.9 | 32.28 | |

**Note:**

# PSMs (the total number of identified peptide spectra matched for the protein)

FDR Confidence Combined was high for all proteins.

Exp. q-value: Combined was between 0-0.007
