# Supplementary material for: Dual roles of the conditional extracellular vesicles derived from Pseudomonas aeruginosa biofilms: Promoting and inhibiting bacterial biofilm growth
Source: Biofilm. 2024 Feb 6;7:100183. doi: 10.1016/j.bioflm.2024.100183 (PMC10876606; doi:10.1016/j.bioflm.2024.100183)
Supplement: Multimedia component 4 [file mmc4.docx]

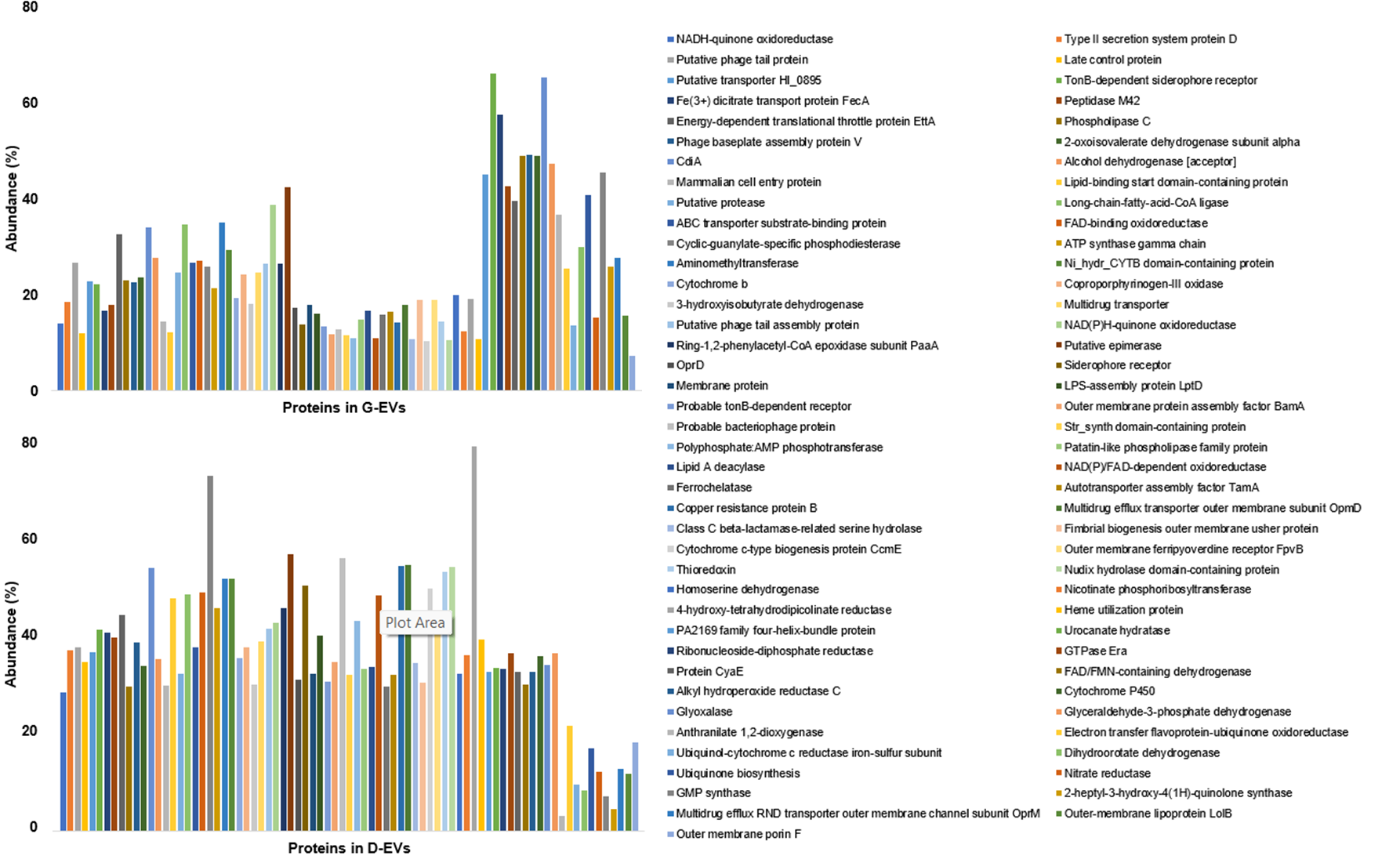


**Fig. S5. Proteomic profiles of 79 surface and cytoplasmic proteins that are shared by both D-EVs and G-EVs with different abundances.**
